# Altered acetylcholine modulations and corticoaccumbal pathway in P11-linked social dysfunction

**DOI:** 10.1038/s41380-025-03324-2

**Published:** 2025-11-03

**Authors:** Daniel Dautan, Anderson Camargo, Niclas Branzell, Valentina I. Brioschi, Daniel Doyon, Elisa Covatta, Roberta Marongiu, Michael Kaplitt, Karima Chergui, Xiaoqun Zhang, Per Svenningsson

**Affiliations:** 1 Department of Clinical Neuroscience, Karolinska Institute, Stockholm, Sweden; 2 Department of Neurological Surgery, Weill Cornell Medicine, New York, NY USA; 3 Department of Physiology and Pharmacology, Karolinska Institute, Stockholm, Sweden

**Keywords:** Neuroscience, Molecular biology

## Abstract

Social relationships rely on the willingness to interact with others and the ability to interpret their emotional cues. Major depressive disorder (MDD) often leads to dysfunctional social interactions, marked by reduced social motivation and difficulties in recognizing emotions, yet these issues remain inadequately explored despite their significant impact on quality of life. These social behaviors, interconnected through the corticoaccumbal pathway, balance anxiety and social interaction, but the underlying mechanisms remain poorly understood. Notably, the calcium-binding protein S100A10 (also known as P11), which is dysregulated in MDD patients and influences the response to antidepressants, is prominently expressed in brain structures involved in social and emotional processing. Here, we demonstrate that chronic restraint stress alters P11 expression along the corticoaccumbal circuit. Additionally, our genetic model, P11-knockout mice, exhibit depression-like behavior, including a reduction of social motivation and impaired recognition of conspecific emotions. Using in vivo and ex vivo electrophysiology, we reveal that P11 expression modulates the response of the corticoaccumbal pathway, influencing the balance between anxiety and social interaction, as well as emotion recognition by regulating dopamine and acetylcholine release in the accumbens. Interestingly, we pinpoint the role of different cholinergic structures in anxiety, social motivation, and emotion recognition. Finally, we show that the prosocial compound oxytocin and social buffering therapy were able to rescue the socially impaired behaviors following chronic stress or P11 ablation, opening new avenues for potential treatments.

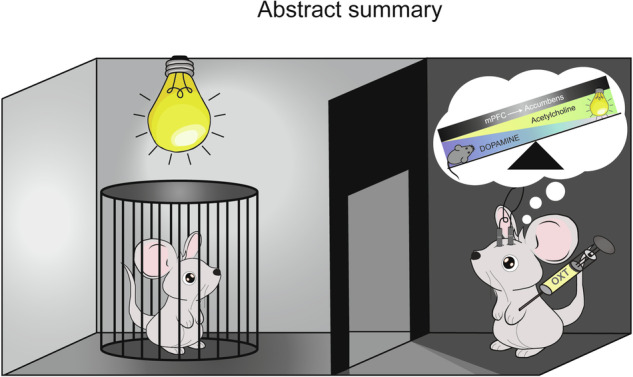

## Introduction

Major depressive disorder (MDD) is a prevalent condition affecting millions worldwide [1], with symptoms extending beyond low mood and suicidal thoughts to include motivation loss, sleep problems, and cognitive issues [2, 3]. These MDD features contribute to poor personal relationships, nonverbal social perception and social functions [4–6]. Both monoamine- and glutamate-based antidepressants have been primarily developed to counteract lowered mood and suicidal thoughts [3, 7], but these therapies rarely consider effects on emotion processing and social interactions [8, 9]. Previous work has indicated that interneurons as well as projection neurons of the medial prefrontal cortex (mPFC) play a key role in balancing anxiety, emotions and the need for social interaction [10–12]. However, the influence of chronic stress paradigms and the molecular mechanisms underlying dysfunctional emotion discrimination in depression-like states remain poorly understood.

A protein that regulates depression-like states and mediates antidepressant responses in rodents is P11 (also named S100A10) [13, 14]. P11-expression is reduced in limbic areas of post-mortem human brain from patients diagnosed with MDD and/or suicide victims [13, 15–17]. Knocking out P11 (P11-KO) induces depressive and anxiety-related behaviors in mice [18], while antidepressant treatment elevates P11 levels in specific brain regions [13, 19]. Conditional KO mouse models have highlighted the role of P11 in depression and antidepressant efficacy, particularly in cholinergic interneurons of the nucleus accumbens (Nac) [16, 20, 21]. While these findings suggest that mutant P11 mouse models provide valuable tools to model depression-like phenotypes, the role of P11 in social functioning has not been examined.

Because MDD symptomatology is complex, multiple brain structures and neurotransmitters have been implicated in its pathophysiology [3, 22]. For example, in Nac, dopamine release is under modulation of cholinergic interneurons to modulate motivation [23, 24] as well as from acetylcholine release from the pedunculopontine nucleus (PPN) and the laterodorsal tegmental nucleus (LDT) to regulate cognitive flexibility and motivation [25–29], which are all impaired in depression-like models [30, 31]. Interestingly, LDT neurons send direct projections to the Nac to modulate reward-related behaviors [28, 32].

Given this background, we hypothesized that impairments of dopamine/acetylcholine balance in corticoaccumbal circuits may be involved in socially linked depression- and anxiety-related behaviors. To address this hypothesis, this study used chronic stress and P11 mutant mice and investigated these models in emotion discrimination, anxiety, and prosocial behavioral tests along with in vivo and ex vivo electrophysiology, dual-sensors photometry, and pharmacological strategies.

## Materials and methods

### Animals

Adult Wild-Type (WT, C57Bl6, 8-12 weeks), P11-WT, constitutive P11-knockout (P11-KO), constitutive P11-knockdown (P11-HET) or conditional P11 knockout in choline acetyltransferase neurons (ChAT-cP11-KO) and their respective controls (P11^flx/ flx^) were generated on-site as previously described [13, 20]. All animals were maintained in home cages with cage mates of similar genetic backgrounds (WT, P11-KO, P11-HET, and P11-WT) or mixed (ChAT-cKO-P11, P11^flx/ flx^). Animals had ad libitum access to food and water across all experiments and were maintained on a strict 12 h light-dark cycle. Both males and females were used, and their genetic background was confirmed by PCR. All experiments were approved by the Karolinska Institute Ethical Committee (3218-2022) according to the Swedish guidelines and conducted in accordance with the European Communities Council Directive of 24 November 1986 (86/609/EEC). Mice were housed in temperature- and humidity-controlled rooms (20 °C, 53% humidity).

### Experimental design

Details for experiments including chronic restraint protocol, drug administration, immunostaining, behavioral testing, virus administration, fiber photometry experiments as well as in vivo and ex vivo electrophysiology are included as supplementary materials.

### Statistical analyses and data sharing

All data are represented as mean ± SEM with individual data points or as scatterplot. Appropriate parametric tests (two-tailed unpaired or paired Student’s t-tests) were chosen based on the population distribution. 1-way ANOVA was used when comparing one variable of several population samples. 2 or 3-way ANOVA was used when analyses accounted for 2 or more distinct variables. Post-hoc Bonferroni analysis results are mentioned in each figure using *. The test used, the number of samples, and the P-values can be found in the results section or the supplementary statistical file.

## Results

### A chronic restraint stress protocol induces anxiety and impairs emotional discrimination behaviors

**Fig. 1:**
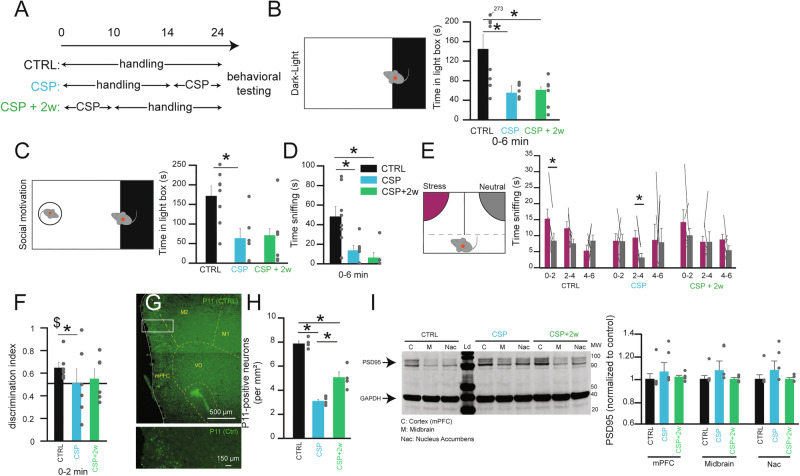
Social motivation and emotion discrimination in chronically stressed mice. (**A**) Design of the behavioral experiments for chronic restrain stress protocol (CSP) and delay of 2 weeks following chronic restrain stress protocol (CPS+2w). (**B**) Time in the light zone during the entire dark-light box test (0–6 min) for CTRL (black), CSP (blue), and CSP+2w (green). 1-way ANOVA F(2,17) = 5.75, *P* = 0.0123; post hoc CTRL versus CSP *P* = 0.04, CTRL versus CSP+2w *P* = 0.0261. (**C**) Time in the light zone during the entire dark-light box test with a WT-conspecific introduced in the light area (0–6 min) for CTRL (black), CSP (blue), and CSP+2w (green). 1-way ANOVA F(2,17) = 5.16, *P* = 0.01769; post hoc CTRL versus CSP *P* = 0.037, CTRL versus CSP+2w *P* = 0.052. (**D**) Time sniffing the WT stimulus during the entire dark-light box test with a WT-conspecific introduced in the light area (0–6 min) for CTRL (black), CSP (blue), and CSP+2w (green). 1-way ANOVA F(2,17) = 8.41, *P* = 0.00288, post hoc CTRL versus CSP *P* = 0.0194, CTRL versus CSP+2w *P* = 0.004718. (**E**) Time sniffing the stress (purple) or neutral (grey) stimulus in the entire emotional discrimination test for CTRL (black), CSP (blue), and CSP+2w (green). paired t-tests CTRL 0–2 min *P* = 0.001, 2–4 min *P* = 0.2018, 4–6 min *P* = 0.2979, 0–6 min *P* = 0.01164; CSP 0–2 min *P* = 0.9554, 2–4 min *P* = 0.0133, 4–6 min *P* = 0.9309, 0–6 min *P* = 0.506; CSP+2w 0–2 min *P* = 0.3189, 2–4 min *P* = 0.9946, 4–6 min *P* = 0.1595, 0–6 min *P* = 0.2484. (**F**) Discrimination index between stress and neutral stimulus during the 1^st^ two min for CTRL (black), CSP (blue), and CSP+2w (green). 0–2 min, 1-way ANOVA F(2,15) = 6.0456, *P* = 0.012, post hoc CTRL versus CSP *P* = 0.013, CTRL versus CSP+2w *P* = 0.112, CSP versus CSP+2w *P* = 0.75. (**G**) Immunostaining for P11 protein expression in the frontal cortex. (**H**) Number of P11-positive cells per mm^2^ in the frontal cortex for CTRL (black), CSP (blue), and CSP+2w (green). 1-way ANOVA F(2,9) = 68.368, *P* = 0.000004, post hoc CTRL versus CSP *P* = 0.000003, CTRL versus CSP+2w *P* = 0.000232, CSP versus CSP+2w *P* = 0.002858. (**I**) Western blot of fresh brain tissue collected in the frontal cortex (C), ventral midbrain (M), and the nucleus accumbens (Nac) immediately after emotion discrimination and quantification of the protein PSD95 (normalized to the loading control GAPDH) for CTRL (black), CSP (blue), and CSP+2w (green). 2-way ANOVA group effect F(2,27) = 4.7, *P* = 0.0176, structure effect F (2,27) = 0.011, *P* = 0.989, interaction effect F(4,27) = 0.043, *P* = 0.996, post hoc on group effect CTRL versus CSP *P* = 0.0495, CSP versus CSP+2w *P* = 0.0318, CTRL versus CSP+2w *P* = 0.99. Data are represented as mean ± SEM. Individual data are represented as dots when possible. Lines between histograms are used to represent time in a zone or interaction between conditions. Ld: Ladder. MW: Molecular weight. *P < 0.05. $ *P* < 0.05 in comparison with 0.5 DI.

To induce depression- and anxiety-like states we used repetitive immobilization as a chronic stress protocol (CSP) [33]. Here, we assessed the effects of CSP on social behavior, anxiety, social motivation, and emotion recognition. We first tested the short- and long-lasting effects of CSP by testing a group of mice immediately after CSP (*n* = 6, CTRL, *n* = 8) or 2 weeks after the CSP protocol (CSP+2w, *n* = 6, Fig. 1A). In the dark-light box, a test commonly used for anxiety phenotypes, we found that CSP and CSP+2w were sufficient to reduce the time in the light area (Fig. 1B). The introduction of a conspecific in the light area, to test social motivation was not sufficient to rescue the phenotype in both CSP and CSP+2w (Fig. 1C) as well as their social interaction (Fig. 1D). The presence of an object (Fig. S1A) or familiar food (Fig. S1B) similarly did not improve their anxiety-phenotypes. We next tested the effect of CSP on emotion discrimination (EDT) (Fig. 1E). While control animals were able to discriminate the stress states of a demonstrator mouse, the discrimination was not present in either CSP or CSP+2w (Fig. 1E). An effect was also observed in their discrimination index (Fig. 1F**)**. No significant difference was observed following CSP in discriminating between two neutral stimuli (Fig. S1C), suggesting that social interaction was not impaired by CSP protocols.

### Chronic restraint stress protocol affects the levels of P11, PSD-95, and DAT in the midbrain, nucleus accumbens, and prefrontal cortex

We then tested whether CSP induces molecular changes including P11 expression, dopamine transporter (DAT), and synaptic markers. Immunostaining for P11 (Fig. 1G) revealed a reduced number of cortical neurons expressing P11 in the mPFC (Fig. 1H). Similarly, CSP was associated with a lasting reduction of dopamine release in the striatum [34]. Immunostaining for DAT (Fig. S1D-G) showed a significant effect in CSP+2w mice on normalized DAT immunofluorescence in the nucleus Accumbens shell (NacSh, Fig. S1E), core (NacC, Fig. S1F) as well as the dorsal striatum (Str, Fig. S1G) together with a trend for CSP group. Examination of the post-synaptic marker of excitatory inputs (PSD-95) in the cortex, midbrain, and Nac (*n* = 4 mice per group) revealed a significant increase of PSD95 in all structures after CSP (Fig. 1I). These results suggest a relation between impairment of social phenotypes and molecular alterations in proteins regulating excitatory and dopaminergic inputs between Nac and mPFC.

### P11-KO mice recapitulate the anxiety-related and depressive-like phenotype observed in chronically stressed mice

**Fig. 2:**
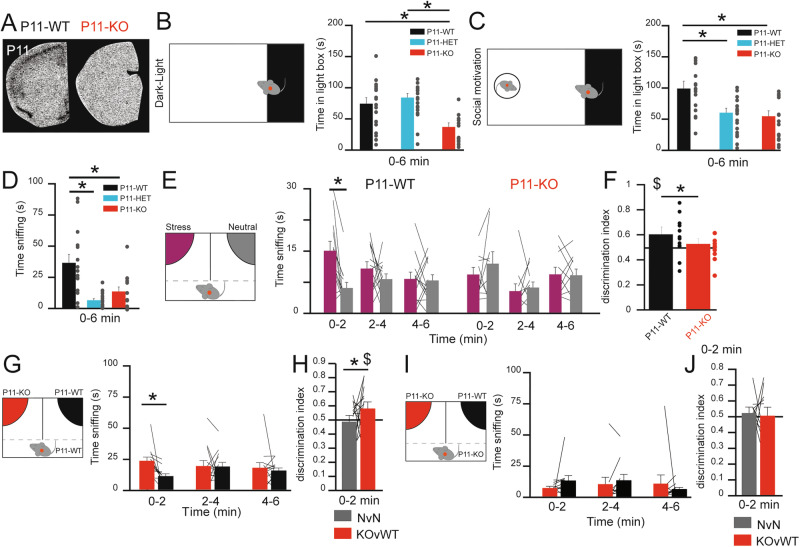
P11-KO mice present similar depressive-like phenotypes as chronically stressed animals. (**A**) In situ hybridization of P11 mRNA expression in the cortex of P11-WT or P11-KO mice. (**B**) Time in the light zone during the entire dark-light box test (0–6 min) for P11-WT (black), P11-HET (blue), and P11-KO (red). 1-way ANOVA F(2,45) = 6.004, *P* = 0.004884, post hoc P11-WT versus P11-KO *P* = 0.038, P11-WT versus P11-HET *P* = 0.98, P11-HET versus P11-KO *P* = 0.0043. (**C**) Time in the light zone during the entire dark-light box test with a WT-conspecific introduced in the light area (0–6 min) for P11-WT (black), P11-HET (blue), and P11-KO (red). 1-way ANOVA F(2,45) = 8.4398, *P* = 0.000772, post hoc P11-WT versus P11-KO *P* = 0.0016, P11-WT versus P11-HET *P* = 0.004, P11-HET versus P11-KO *P* = 0.99. (**D**) Time sniffing the WT stimulus during the entire dark-light box test with a WT-conspecific introduced in the light area (0–6 min) for P11-WT (black), P11-HET (blue), and P11-KO (red). 1-way ANOVA F(1,45) = 7.21, *P* = 0.000021, poct hoc P11-WT versus P11-KO *P* = 0.001532, P11-WT versus P11-HET *P* = 0.000019, P11-HET versus P11-KO *P* = 0.7157. (**E**) Time spent sniffing the stress (purple) or neutral (grey) stimulus in the emotional discrimination test for each segment of 2 min (0–2, 2–4, and 4–6 min) for P11-WT (black) and P11-KO (red). paired t-tests P11-WT 0–2 min *P* = 0.0015, 2–4 min *P* = 0.334, 4–6 min *P* = 0.865, 0–6 min *P* = 0.0117; P11-KO: 0–2 min *P* = 0.372, 2–4 *P* = 0.668, 4–6 min *P* = 0.8948, 0–6 min *P* = 0.4369. (**F**) Discrimination index between stress and neutral stimulus during the 1^st^ two min for P11-WT (black) and P11-KO (red). unpaired t-tests *P* = 0.0416. (**G**) Time spent sniffing P11-KO (red) or P11-WT (black) stimulus for each segment of 2 min (0–2, 2–4, and 4–6 min) for P11-WT observer. paired t-tests 0–2 min *P* = 0.0071, 2–4 min *P* = 0.9246, 4–6 min *P* = 0.6303, 0–6 min *P* = 0.0404. (**H**) Discrimination index between the Neutral versus Neutral (NvN) and the P11-KO versus P11-WT (KOvWT) discrimination test over the 1^st^ two min for a P11-WT observer. paired t-tests *P* = 0.00411. (**I**) Time spent sniffing P11-KO (red) or P11-WT (black) stimulus for each segment of 2 min (0–2, 2–4, and 4–6 min) for P11-KO observer. paired t-tests 0–2 min *P* = 0.258, 2–4 min *P* = 0.3713, 4–6 min *P* = 0.5789, 0–6 min *P* = 0.87. (**J**) Discrimination index between the Neutral versus Neutral (NvN) and the P11-KO versus P11-WT (KOvWT) discrimination test over the 1^st^ two min for a P11-KO observer. paired t-tests *P* = 0.64. Data are represented as mean ± SEM. Individual data are represented as dots when possible. Lines between histograms are used to represent time in a zone or interaction between conditions. *P < 0.05. $ *P* < 0.05 in comparison with 0.5 DI.

In P11-KO mice (Fig. 2A), we observed a similar reduction of DAT levels in striatal regions (including dorsal and ventral; Fig. S2A-B), suggesting that ablation of P11 resembles CSP effects. We first tested whether a partial reduction (P11-HET^+/−^, *n* = 18) or global knockout (P11-KO^−/−^, *n* = 12) of P11 was sufficient to increase anxiety behaviors in the Dark Light box DLB (Fig. 2B). P11-WT^+/+^ (*n* = 18) were used as control. We found that a global reduction of P11 expression, but not partial, induces anxiety behavior (Fig. 2B). Introducing a conspecific in the light area did not improve the anxiety phenotypes in P11-KO mice, and even impaired social motivation in P11-HET mice as observed by a decrease in the time spent in the light area (Fig. 2C) and in the time sniffing (Fig. 2D). Similarly, introducing an object (Fig. S2C**)** or familiar food **(**Fig. S2D) did not improve anxiety phenotypes in P11-KO and P11-HET mice. We next tested whether P11-KO mice display a significant impairment of emotion discrimination like the one observed in the CSP mice. In the presence of a stressed mouse, we found significant discrimination in P11-WT mice, a response not observed in P11-KO mice (Fig. 2E). Moreover, we observed a significant reduction in the discrimination index in P11-KO mice (Fig. 2F). As expected, we did not observe any differences in the discrimination between two neutral stimuli (Fig. S2E). In addition, we tested P11-WT (*n* = 11) and P11-KO (*n* = 13) mice in the open field, where one side was randomly exposed to drops of vanilla extract (Fig. S2F). Using this olfactory test, we found that olfactory discrimination is not impaired in P11-KO animals and thus confirm that social alteration is linked with the emotional status of the animals. These findings suggest that P11 haploinsufficiency (i.e. P11-HET) is sufficient to impair social motivation, but does not induce anxiety, while a complete loss of P11 (P11-KO) impacts all phenotypes.

To confirm that P11-KO mice display an anxiety-like phenotype like CSP-exposed mice, and to distinguish between P11-WT and P11-KO mice, we tested P11-WT mice (*n* = 13) in the EDT (Fig. 2G, H). Interestingly, we found that P11-WT mice display a preference towards the side paired with P11-KO mice (Fig. 2G) as determined by the discrimination index (Fig. 2H). In sharp contrast, when tested in the same conditions, P11-KO mice (*n* = 10) did not show any significant preference for either stimulus mice (Fig. 2I) or the discrimination index (Fig. 2J). These data suggest that not only P11-KO mice display a basal phenotype that can be discriminated by P11-WT animals, but such discrimination is also impaired in P11-KO mice.

### Dysregulation of the mPFC and Nac pathway in P11-KO mice

**Fig. 3:**
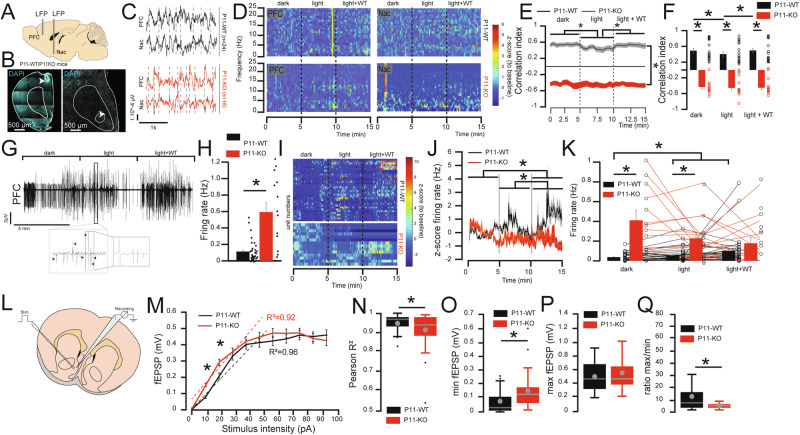
Expression of P11 is required for cortical adaptation to a new environment in the presence of cage mates. (**A**) Illustration of the simultaneous recording of local field potential (LFP) in the mPFC and the Nac of P11-WT and P11-KO mice that allows comparison of the correlation between both signals. (**B**) Confocal images of the location of the LFP electrodes in the mPFC and the Nac. (**C**) Example traces of the LFP in the mPFC and the Nac in P11-WT (black, top) and P11-KO (red, bottom). (**D**) Examples of the colored raster plot of the z-score Power spectrum in 2 mice, P11-WT (top) and P11-KO (bottom), during the dark (5 min), light (5 min), and with same-sex conspecific mice (light+WT, 5 min) stages in the mPFC (left) and the Nac (right). (**E, F**) Correlation index and average correlation index between the LFP of mPFC and Nac during the dark, light, and light+WT conditions within a 30 s bin size in P11-WT (black) and P11-KO (red) mice. 2-way ANOVA stage effect F(2,114) = 0.514, *P* = 0.599, genotype F(1,114) = 354.13, *P* = 0.000001, interaction F(2,114) = 0.28, *P* = 0.75. Poshoc analyses: P11-WT: dark to light: *P* = 0.0136; light to light+WT *P* = 0.0293; light+WT to dark: *P* = 0.121. P11-KO: dark to light: *P* = 0.5447; dark to light+WT: *P* = 0.5267, light to light+WT *P* = 0.78. P11-WT versus P11-KO: in dark: *P* = 0.00001, in Light: *P* = 0.00001 and light+WT: *P* = 0.00001. (**G**) Example of filtered LFP in the mPFC of P11-WT mice during the dark (5 min), light (5 min), and light+WT (5 min) stages showing the different putative single units. (**H**) Average firing rate of mPFC putative single units during the 15 min recordings in P11-WT (black) and P11-KO (red). unpaired t-test *P* = 0.000001. (**I**) Colored raster plot of the z-score firing rate of mPFC putative single units during the dark (5 min), light (5 min), and the light+WT (5 min) stages in all P11-WT (top) and P11-KO (bottom). (**J**) z-score normalization of the firing rate of mPFC putative single units recorded during the dark (5 min), light (5 min), and light+WT (5 min) behavior in P11-WT (black) and P11-KO (red). 2-way ANOVA group effect F(1,144) = 21.75, *P* = 0.0000007, time effect F(2,144) = 2.61, *P* = 0.0769, interaction F(2,144) = 9.34, *P* = 0.000152, post hoc P11-WT dark versus light *P* = 0.5704, P11-WT dark versus light + WT *P* = 0.000001, P11-WT light versus light+WT *P* = 0.006252. P11-KO: *P* > 0.05. (**K**) Average firing rate of mPFC putative single units during the 5 min dark (left), light (middle) and light+WT (right) in P11-WT (black) and P11-KO (red). 2-way ANOVA stage effect F(2,144) = 2.98, *P* = 0.0537, genotype F(1,144) = 41.02, *P* = 0.000001, interaction F(2,144) = 6.94, *P* = 0.0013, post hoc on dark stage: WT versus KO *P* = 0.000001, light stage: WT versus KO *P* = 0.014519; light+WT stage: WT versus KO *P* = 0.149, post hoc on genotype P11-WT: dark versus light *P* = 0.002, dark versus light+WT *P* = 0.021, light versus light+WT *P* = 0.102, P11-KO: *P* > 0.05. (**L**) Schematic of the recording of fEPSP/PS in P11-WT and P11-KO. (**M**) Response curve in P11-WT (black) and P11-KO (red) to progressive increase of stimulation intensity. The average trendline between 10 and 50 µA is represented together with the Pearson correlation index (R^2^). 10 µA: *P* = 0.00305; 20 µA: *P* = 0.00648; other P > 0.05. (**N**) Box-plot of individual R^2^ index for each recording between 10 and 50 µA stimulation. unpaired t-test *P* = 0.0498. (**O**) Box-plot of the minimum response to electrical stimulation (10 or 20 µA) for each recording. unpaired t-test *P* = 0.00305. (**P**) Box-plot of the maximum response to electrical stimulation for each recording (50 to 90 µA). unpaired t-test *P* = 0.478. (**Q**) Box-plot of the ratio between maximum and minimum response for each recording. Unpaired t-test *P* = 0.00183. Data are represented as mean ± SEM. Box plot represent the 75- and 95-percentile, mean and SEM as well as outlier data. Individual data are represented as dots when possible. Lines between histograms are used to represent changes in firing rate across conditions. *P < 0.05.

The mPFC plays a crucial role in social motivation and emotion recognition [11]. Moreover, P11 is expressed in many structures including Nac and mPFC (Fig. S3A) [35]. Here we tested to which extent the corticoaccumbal pathway and P11 participate in social motivation and emotion. Accordingly, we recorded local field potential (LFP) in P11-WT mice (*n* = 24) and P11-KO mice (*n* = 16) during a social behavior task (Fig. 3A). The position of the electrodes within the mPFC or Nac was confirmed postmortem (Fig. 3B, S3B). Simultaneous LFP recording of ipsilateral PFC and Nac allowed to compare both signals (Fig. 3C) as well as their relationship. The signal was collected in the animal’s home-cage during a behavior task combining light-OFF (dark, 5 min), light-ON (light, 5 min), and the introduction of a conspecific (light+WT, 5 min, Fig. 3D), a task specifically designed to test anxiety and the ability to cope with a conspecific. Individually, we found an increase of Nac and mPFC high-frequency activity in the light and light+WT context (Fig. 3D) in P11-WT mice, while for P11-KO mice the increase was mostly in the low-frequency range (Fig. 3D).

When comparing the correlation between both signals, we found a positive correlation between mPFC and Nac in P11-WT mice during the control phase (dark, mean correlation index 0.50 + /-0.017, max index: 0.87 and min index: 0.202, Fig. 3E, F). When the light was turned on the correlation index decreased (light, mean index 0.4191 + /-0.0193, max: 0.887, min: -0.058) and returned immediately after the introduction of P11-WT mice (mean index 0.4841 + /-0.183, max 0.8994, min 0.2061, Fig. 3E, F). In sharp contrast, we observed no variation of signal in P11-KO mice in the light condition or the light+WT (Fig. 3E, F). Curiously, we also found the opposite effect in P11-KO mice where the correlation index was in sharp opposition with that of P11-WT mice during the dark (mean correlation -0.435 + /-0.02, max: 0.212, min: -0.697), the light (mean correlation -0.454 + /-0.019, max: 0.1494, min: -0.879) and the light+WT (mean correlation -0.45 + /-0.0167, max 0.039, min –0.673) conditions (Fig. 3E, F) suggesting that the connectivity between mPFC and Nac encodes anxiety and is strongly impaired in P11-KO mice.

To confirm the involvement of mPFC neurons, we recorded single-unit activity in freely moving mice during the same behavioral test (Fig. 3G). We found a significantly higher firing rate (FR) in P11-KO mice (mean FR 0.564 + / + 0.1 Hz, max 1.792 Hz, min 0.01859 Hz) compared to P11-WT mice (mean FR 0.106 + /-0.02, max 0.53, min 0.0024, Fig. 3H) during the baseline period. We next tested the mice in the same behavior as above (dark, light, light+WT, Fig. 3I). We found that the average z-score FR varies during the different behavior tasks (Fig. 3J). We observed no significant variation in P11-KO mice during the different stages (Fig. 3J). In addition, we found a similar result by looking at the FR where P11-WT FR increases in Light and Light+WT while the one of P11-KO does not change during the different stages (Fig. 3K). These data suggest a hyperactivation in vivo of the mPFC neurons projecting to Nac in P11-KO mice accompanied by an inverted electrophysiological encoding of social interaction between P11-WT and P11-KO mice and thus confirm previous observation [11].

To see how the absence of P11 perturbates the corticoaccumbal pathway, we recorded ex vivo the fEPSP/PS evoked by electrical stimulations of progressively increasing intensities (10–90 pA) and measured the response amplitude (Fig. 3L) in P11-WT (*n* = 3, 34 recordings) or P11-KO (*n* = 3, 34 recordings) mice intending to activate cortical projection in Nac. When comparing the response to electrical stimulation we found a significant increase in the response amplitude in P11-KO mice compared to P11-WT mice at 10pA and 20pA (Fig. 3M), although at higher intensities the response was not significantly different. We next compared the Pearson correlation between the amplitude and the stimulation intensity between 10 and 50pA (Fig. 3N) and found a lower correlation in P11-KO mice (Fig. 3N). Finally, we found a significant difference in the minimum response (Fig. 3O), but not the maximum response amplitude (Fig. 3P). Taken together, this led to a significant difference in the max/min ratio between the genotypes (Fig. 3Q). These data suggest an impairment of the corticoaccumbal circuit in P11-KO mice that is unrelated to behavioral conditions.

### Dopamine and acetylcholine release in VTA/Nac encode context-dependent anxiety

Motivation is commonly paired with dopamine activity from the VTA [36–40] and its projections to Nac [41, 42]. The LDTg constitute the main cholinergic inputs to VTA and also provides inputs to Nac [27, 29, 32] to modulate motivation [26, 27]. To characterize dopamine/acetylcholine release during social behavior, and particularly the impairment observed in P11-KO mice, we used dual-wavelength fiber photometry recordings of dopamine (AAV-rGRAB-DA) and acetylcholine (AAV-ACh-SnFr) release in P11-WT (*n* = 6 per group) and P11-KO mice (*n* = 6 per group) [24].

**Fig. 4:**
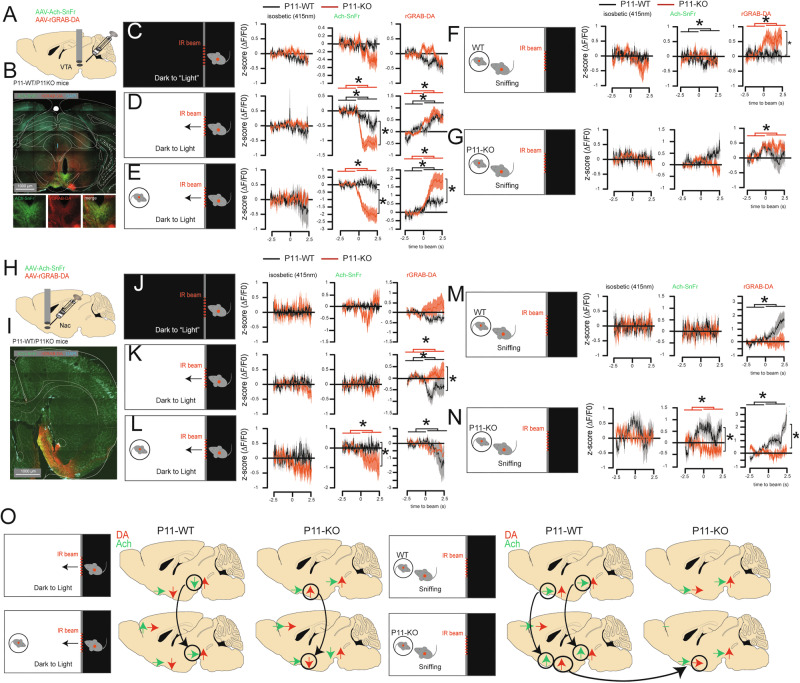
Balance between acetylcholine- and dopamine-release in anxiety and social motivation conditions. (**A,** **B**) Injection of dopamine- (AAV-rGRAB-DA, red) and acetylcholine-sensor (AAV-Ach-SnFr, green) virus in VTA of P11-WT or P11-KO mice allows recording of dopamine and acetylcholine release in medial VTA using fiber photometry. (**C-G**) Variation of the GFP-isosbestic control channel (left, *P* > 0.05), acetylcholine (middle), and dopamine release (right panel) in 5 s windows surrounding entry to the light area in a light OFF condition (**C**, Ach-sensor: paired t-tests baseline versus entries, P11-WT *P* = 0.1547, P11-KO *P* = 0.05112, DA-sensor: paired t-tests P11-WT *P* = 0.365, P11-KO *P* = 0.544), light ON (**D** Ach-sensor: paired t-tests P11-WT *P* = 0.041, P11-KO *P* = 2.10^-9, genotype unpaired t-test *P* = 0.0061; DA-sensor: paired t-tests P11-WT *P* = 0.0004, P11-KO *P* = 8.10^-6, genotype unpaired t-test *P* = 0.5227) and with a conspecific of the same sex animal in the light area (**E** Ach-sensor: paired t-tests P11-WT *P* = 0.6142, P11-KO *P* = 4.10^-6, genotype unpaired t-test *P* = 0.004; DA-sensor: paired t-tests P11-WT *P* = 8.10^-5, P11-KO *P* = 3.10^-5, genotype unpaired t-test *P* = 0.0416) in P11-WT (black) and P11-KO (red) mice. Variation of the GFP-isosbestic control channel (left), acetylcholine (middle, paired t-test P11-WT interacting with P11-WT: *P* = 0.0224; P11-WT interacting with P11-KO: *P* = 0.0776; P11-KO interacting with P11-WT *P* = 0.6402, P11-KO interacting with P11-KO *P* = 0.2471), and dopamine release (right panel, paired t-test P11-WT interacting with P11-WT: *P* = 0.8482; P11-WT interacting with P11-KO: *P* = 0.7933; P11-KO interacting with P11-WT *P* = 0.0027, P11-KO interacting with P11-KO *P* = 0.0144) in a 5 s window surrounding sniffing of P11-WT (**F**) and P11-KO (**G**). (**H, I**) Injection of dopamine- (Aav-RgRAB-DA) and acetylcholine-sensor (AAV-Ach-SnFr) virus in the Nac (**H**) of P11-WT or P11-KO mice allow recording of dopamine and acetylcholine release in Nac using fiber photometry (**I**). (**J-N**) Variation of the GFP-isosbestic control channel (left), acetylcholine (middle), and dopamine release (right panel) in a 5 s window surrounding entry to the light area in a light OFF condition (**J**), light ON (**K** Ach-sensor: paired t-tests P11-WT *P* = 0.105, P11-KO *P* = 291, genotype unpaired t-test *P* = 0.1792; DA-sensor: paired t-tests P11-WT *P* = 0.004, P11-KO *P* = 0.028, genotype unpaired t-test *P* = 0.0003) and with a conspecific of the same sex animal in the light area (**L**, Ach-sensor: paired t-tests P11-WT *P* = 0.468, P11-KO *P* = 0.041, genotype unpaired t-test *P* = 0.0119; DA-sensor: paired t-tests P11-WT *P* = 0.043, P11-KO *P* = 0.262, genotype unpaired t-test *P* = 0.1299) in P11-WT (black) and P11-KO (red) mice. Variation of the GFP-isosbestic control channel (left), acetylcholine (middle, paired t-test P11-WT interacting with P11-WT: *P* = 0.1561; P11-WT interacting with P11-KO: *P* = 0.4121; P11-KO interacting with P11-WT *P* = 0.93, P11-KO interacting with P11-KO *P* = 0.011) and dopamine release (right panel, paired t-test P11-WT interacting with P11-WT: *P* = 0.0407; P11-WT interacting with P11-KO: *P* = 7.10^-8; P11-KO interacting with P11-WT *P* = 0.8592, P11-KO interacting with P11-KO *P* = 0.43) in a 5 s window surrounding sniffing of P11-WT (**M**) and P11-KO (**N**). (**O**) Schematic representation of the variation of dopamine (red) and acetylcholine (green) release during the dark-light area, dark-light social, and sniffing. Data are represented as z-score normalization of the ΔF/F0. Statistical analyses are between the baseline versus entry time (top) or between the P11-WT and P11-KO (right side). *P < 0.05.

Following injection of AAVs in VTA (Fig. 4A-G, S4A-D) or Nac (Fig. 4H, N, S4E-H) and optic fiber implant above the injection site (Fig. 4B, I) we collected locally released dopamine [26] and acetylcholine signal in VTA [27, 28] or Nac [32].

The mice were recorded in a custom-made DLB, in which crossing the door to (Fig. 4C-N) or from (Fig. S4A-H) the light area was used for signal scoring. The average signals during the 2.5 s baseline and 2.5 s following entries were compared across the different conditions, and a second analysis focused on genotype differences following the 2.5 s entries. The protocol consists of 5 different conditions aiming to assess anxiety, social motivation, and emotion discrimination with a complete darkness as baseline (Fig. 4C, J). Across all conditions and both targets, we did not observe any significant changes of signal in the isosbestic channels during all tests (first column, Fig. 4C-G, J-N).

In VTA (Fig. 4C) and Nac (Fig. 4J) during complete darkness (Light-OFF conditions), we found no significant changes, in P11-WT or P11-KO mice, when entering the “light-area” of the signal arising from the Ach- (middle column) or the DA-sensor (right column).

When the light was turned-ON (i.e. testing anxiety), we observed that entering the light area was sufficient to decrease Ach-release in VTA in both P11-WT and P11-KO mice (Fig. 4D) whereas exiting the light area was paired with a reduction of the signal in P11-WT, but not in P11-KO, mice (Fig. S4C) suggesting that Ach-signal in VTA encodes anxiety as well as relief. In sharp contrast, DA-sensor activity increases in both P11-WT and P11-KO mice when entering the light area (Fig. 4D) but decreases when exiting when exiting (Fig. S4C). When comparing genotype effects, we found that Ach-sensor signal decrease when entering the light area was stronger in P11-KO mice while DA-sensor increase was similar between genotypes (Fig. 4D). As DA-release in VTA participates in inhibition of DA-neuron activity [26] our data suggest that anxiety conditions might reduce DA-neurons firing rates.

In Nac, during light-ON condition, we found no significant modification of Ach-release when entering or exiting the light area (Fig. 4K, S4G) while DA-sensor signal decreased in P11-WT but increased in P11-KO mice when entering the light area (Fig. 4K) with a significant difference between genotypes. However, when the animal exited the light area, we observed an increase of DA-sensor signal in P11-KO, but not in P11-WT, mice with a significant difference between genotypes (Fig. S4G). Altogether, these results suggest that DA release in both VTA and Nac encodes different context-dependent anxiety.

### Dopamine and acetylcholine release in VTA/Nac encode social motivation

We next tested the introduction of a P11-WT conspecific in the light area, and recorded signal either in the VTA (Fig. 4E, S4D) or Nac (Fig. 4L, S4H) to estimate the effects of social motivation. In VTA, we found that when the animal entered the light area, Ach-sensor activity decreased in P11-KO, but not in P11-WT, mice with a significant difference between genotypes, while DA-sensor signal increased in both genotypes with a stronger effect in P11-KO mice (Fig. 4E). Similarly, when the animals exited the light area, we found a decrease in Ach-sensor signal in P11-WT mice while it increased in P11-KO mice with a significant genotype difference (Fig. S4D) and that DA-sensor activity decreased in P11-KO, but not in P11-WT, mice, with no genotype difference (Fig. S4D).

In Nac, following the introduction of a P11-WT mouse, we found a decrease in the Ach-sensor signal when entering the light area as well as an increase when exiting the area in P11-KO, but not in P11-WT, mice with a significant genotype difference (Fig. 4L, S4H). Interestingly, the DA-sensor decreased in P11-WT mice following the entry (Fig. 4L) or exit (Fig. S4H) of the light area, effects that were not observed in P11-KO mice (Fig. 4L).

Altogether, these photometry results suggest that the levels of Ach and DA release in the VTA are influenced by the emotional status of the conspecific mice placed in the light area. In contrast, since the effects were similar regardless of whether the stimulus mice were P11-WT or P11-KO, DA and Ach release in the Nac does not appear to be crucial for social motivation.

### Dopamine and acetylcholine release in VTA/Nac encode social interaction and emotion recognition

To see if the effects were similar in the presence of a P11-KO mouse, which displays a basal anxiogenic state, we aligned the signal to the initiation of social interaction with either a P11-WT (Fig. 4F-M) or a P11-KO (Fig. 4G-N) stimulus. In the VTA of P11-WT mice, Ach-sensor signal shortly decreased following sniffing a P11-WT, but not a P11-KO, mouse (Fig. 4F, G). In contrast, no changes were found in Ach-sensor signal in P11-KO mice neither when sniffing a P11-WT nor a P11-KO mouse (Fig. 4F, G). Consequently, there was no difference between P11-WT and P11-KO mice when sniffing a P11-WT or a P11-KO mouse (Fig. 4F, G). In contrast, the DA-sensor signal did not change in P11-WT mice when interacting with P11-WT or P11-KO (Fig. 4F, G) mice. However, the same signal increased in P11-KO mice following interaction with a P11-WT and a P11-KO mouse.

In Nac of P11-WT mice, we found that the Ach-sensor signal did not change in response to interaction with a P11-WT or P11-KO stimulus mice while the same Ach-sensor signal decreased in P11-KO observer mice in response to interaction with P11-KO, but not P11-WT, stimulus mice. Interestingly, Ach-signal increased in P11-WT mice in anticipation of interaction with P11-KO, but not P11-WT, mice (Fig. 4N).

There was a significant increase in DA-sensor signals in P11-WT mice both in the presence of P11-WT and P11-KO mice (Fig. 4M, N). This was not found in P11-KO mice regardless of whether the interaction was with a P11-WT or a P11-KO mouse (Fig. 4M). There were significant differences in DA-sensor signals between P11-WT and P11-KO mice in the presence of a P11-KO stimulus but not P11-WT (Fig. 4M, N). These results suggest that, independently of their emotional states, the presence of a stimulus is sufficient to buffer anxiety at the level of DA-release in VTA. Interestingly, Ach-release in VTA seems to encode more specifically the emotional states of the stimulus. In Nac, we found that Ach-signal seems to encode the anticipation to interaction with an emotionally altered stimulus, while DA-signal encodes social interaction independently of the emotional states of the stimulus (Fig. 4O).

### P11 in cholinergic neuronal subtypes plays a role in anxiety- and depression-associated social phenotypes

**Fig. 5:**
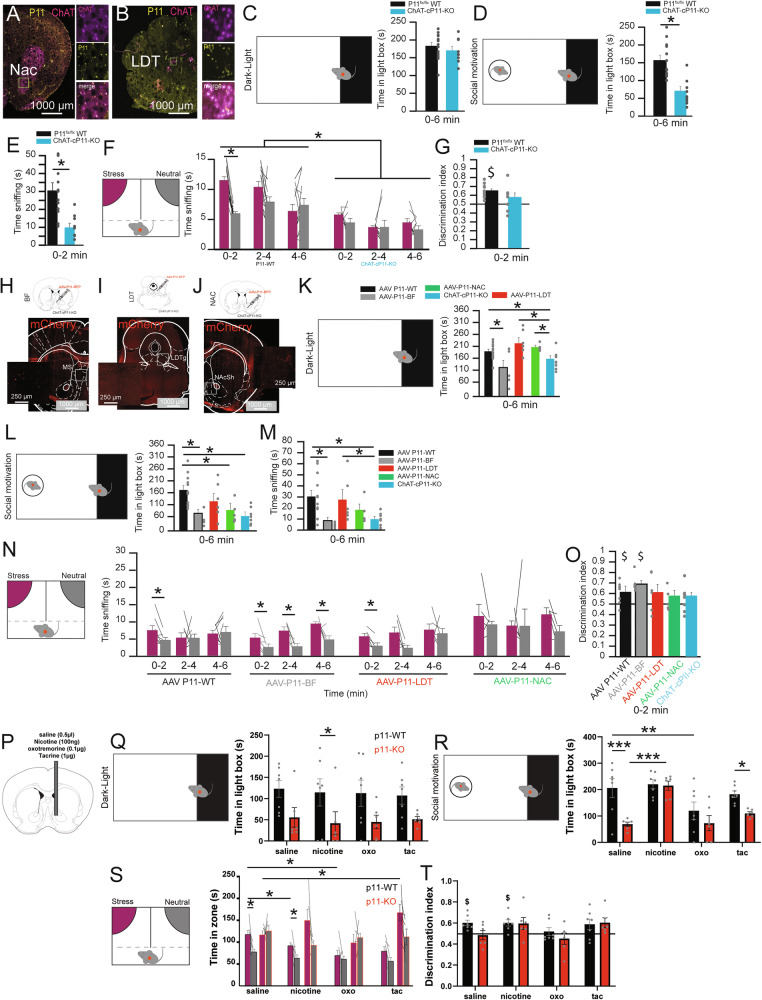
Cholinergic neuronal subtype-specific expression of P11 is involved in specific depression- or anxiety-like phenotypes. (**A, B**) Expression of P11-RNA level in cholinergic interneurons (ChAT) of the Nac (**A**) the brainstem (LDT, **B**) using RNAscope and IHC in P11-WT mice. (**C**) Time in the light zone during the entire dark-light box test (0–6 min) for P11^flx/flx^ WT (black) and, ChAT-cP11-KO (blue). Unpaired t-test *P* = 0.4104. (**D**) Time in the light zone during the entire dark-light box test with a WT-conspecific introduced in the light area (0–6 min) for P11^flx/flx^ WT (black) and ChAT-cP11-KO (blue). Unpaired t-test *P* = 0.0002. (**E**) Time sniffing the WT stimulus during the entire dark-light box test with a WT-conspecific introduced in the light area (0-2 min) for P11^flx/flx^ WT (black) and ChAT-cP11-KO (blue). Unpaired t-test *P* = 0.001. (**F**) Time spent sniffing the stress (purple) or neutral (grey) stimulus in the emotional discrimination test for the entire test (0-6 min) for P11^flx/flx^ WT (black) and ChAT-cP11-KO (blue). Paired t-tests: P11-WT 0-2 *P* = 2.10-6, 2-4 *P* = 0.0594, 4-6 *P* = 0.352, 0-6 *P* = 0.0003. ChAT-cP11-KO: 0-2 *P* = 0.1675, 2-4 *P* = 0.9762, 4-6 *P* = 0.225, 0-6 *P* = 0.289. P11-WT versus ChAT-cP11-KO: stress *P* = 1.83.10^6, neutral *P* = 0.0002. (**G**) Discrimination index between stress and neutral stimulus during the 1^st^ two min for P11^flx/flx^ WT (black) and ChAT-cP11-KO (blue). Unpaired t-tests to 0.5: P11-WT *P* = 1.10-9, ChAT-cP11-KO *P* = 0.0905, P11-WT versus ChAT-cP11-KO *P* = 0.08916. (**H-J**) Representative confocal images of the expression of AAV1-2.CBA.RFP.loxP-P11 injected in ChAT-cP11-KO mice targeting the Basal Forebrain (BF, **H**), LDT (**I**), or NAC (**J**). (**K**) Time in light zone during the entire dark-light box test (0-6 min) for P11-WT mice (injected with AAV, black), ChAT-cP11-KO injected in the BF (grey), the LDT (red) or the NAC (green) or ChAT-cP11-KO (not injected, blue). 1-way ANOVA F(4,33) = 5.06, *P* = 0.002704, post hoc P11-WT versus AAV-P11-BF: *P* = 0.0129, P11-WT versus ChAT-cP11-KO: *P* = 0.0487, ChAT-cP11-KO versus AAV-P11-LDT: *P* = 0.0187, ChAT-cP11-KO versus AAV-P11-NAC: *P* = 0.0178. (**L**) Time in the light zone during the entire dark-light box test with a WT-conspecific introduced in the light area (0-6 min) for P11-WT mice (injected with AAV, black), ChAT-cP11-KO injected in the BF (grey), the LDT (red) or the NAC (green) or ChAT-cP11-KO (not injected, blue). 1-way ANOVA F(4,32) = 6.40, *P* = 0.000662, Post hoc P11-WT versus ChAT-cP11-KO; *P* = 0.00016, P11-WT versus AAV-P11-BF: *P* = 0.03224, P11-WT versus AAV-P11-NAC; *P* = 0.02331, P11-WT versus AAV-P11-LDT: *P* = 0.1864; ChAT-cP11-KO versus AAV-P11-LDT: *P* = 0.6543. (**M**) Time sniffing the WT stimulus during the entire dark-light box test with a WT-conspecific introduced in the light area (0-6 min) for P11-WT mice (injected with AAV, black), ChAT-cP11-KO injected in the BF (grey), the LDT (red) or the NAC (green) or ChAT-cP11-KO (not injected, blue). 1-way ANOVA F(4,33) = 3.43, *P* = 0.0187, post hoc ChAT-cP11-KO versus P11-WT; *P* = 0.00802; AAV-P11-LDT versus ChAT-cP11-KO: *P* = 0.0452; P11-WT versus AAV-P11-BF: *P* = 0.01961, AAV-P11-NAC versus P11-WT; *P* = 0.22734, P11-WT versus AAV-P11-LDT; *P* = 0.7863, ChAT-cP11-KO versus AAV-P11-BF: *P* = 0.7269, ChAT-cP11-KO versus AAV-P11-NAC *P* = 0.11498. (**N**) Time spent sniffing the stress (purple) or neutral (grey) stimulus in the emotional discrimination test for each segment of 2 min (0-2, 2-4, and 4-6 min) for P11-WT mice (injected with AAV, black), ChAT-cP11-KO injected in the BF (grey), the LDT (red) or the NAC (green). paired t-tests P11-WT: 0-2 min *P* = 0.0239, 2-4 min *P* = 0.9818, 4-6 min *P* = 0.7979, 0-6 min *P* = 0.5405; AAV-P11-BF: 0-2 min *P* = 0.047, 2-4 min *P* = 0.027, 4-6 min *P* = 0.030, 0-6 min *P* = 0.000553; AAV-P11-LDT: 0-2 min *P* = 0.0101, 2-4 min *P* = 0.056, 4-6 min *P* = 0.68, 0-6 min *P* = 0.135; AAV-P11-NAC: 0-2 min *P* = 0.44, 2-4 min *P* = 0.97, 4-6 min *P* = 0.063, 0-6 min *P* = 0.164. (**O**) Discrimination index between stress and neutral stimulus during the 1^st^ two min for P11-WT mice (injected with AAV, black), ChAT-cP11-KO injected in the BF (grey), the LDT (red) or the NAC (green). 1-way ANOVA F(1,20) = 2.086, *P* = 0.1341. Compared to 0.5: P11-WT *P* = 0.0025, AAV-P11-BF *P* = 0.0003, AAV-P11-LDT *P* = 0.1551, AAV-P11-NAC *P* = 0.1453. (**P**) Graphical representation of cannula implantation. (**Q**) Time in light zone during the entire dark-light box test (0-6 min) for P11-WT mice (black) and P11-KO (red) 30-min following administration of saline (0.5 µl), nicotine (100 ng/0.5 µl), oxo (0.1 µg/0.5 µl) or tacrine (1 µg/0.5 µl). 2-way RM-ANOVA treatment effect F(3,33) = 0.117, *P* = 0.9493, genotype effect F(1,33) = 10.22, *P* = 0.0085, interaction effect F(3,33) = 0.05, *P* = 0.983. Post hoc nicotine P11-WT versus P11-KO *P* = 0.0374. (**R**) Time in the light zone during the entire dark-light box test with a WT-conspecific introduced in the light area. 2-way RM-ANOVA treatment effect F(3,33) = 9.77, *P* < 0.0001, genotype effect F(1,33) = 13.88, *P* = 0.0033, interaction effect F(3,33) = 2.92, *P* = 0.078. Post hoc saline P11-WT versus P11-KO *P* = 0.0001, nicotine P11-WT versus P11-KO *P* = 0.8731, oxo P11-WT versus P11-KO *P* = 0.1605, tac P11-WT versus P11-KO *P* = 0.0331. P11-WT saline versus oxo *P* = 0.0089. P11-KO saline versus nicotine *P* = 0.0001. (**S**) Time spent in the stress (purple) or neutral (grey) stimulus zone in the emotional discrimination test for the entire period 90-6 min). Paired t-tests: saline P11-WT *P* = 0.0129, P11-KO *P* = 0.69; nicotine P11-WT *P* = 0.0086 P11-KO *P* = 0.1317; Oxo P11-WT *P* = 0.48 P11-KO *P* = 0.58; tac P11-WT *P* = 0.088 P11-KO *P* = 0.0871. P11-WT saline versus nicotine *P* = 0.0303, P11-WT saline versus oxo *P* = 0.0193, P11-KO saline versus tac *P* = 0.0488 (**T**) Discrimination index between stress and neutral stimulus during the entire 6-min P11-WT mice (black) and P11-KO (red) with administration of cholinergic compound. 2-way RM-ANOVA treatment effect F(3,33) = 0.036, *P* = 0.102, genotype effect F(1,33) = 0.024, *P* = 0.102, interaction effect F(3,33) = 0.55, *P* = 0.8467. Compared to 0.5: P11-WT Saline *P* = 0.0021, P11-WT nicotine *P* = 0.0054. Data are represented as mean ± SEM. Individual data are represented as dots when possible. Lines between histograms are used to represent time in a zone or interaction between conditions. *P < 0.05. $ *P* < 0.05 in comparison with 0.5 DI.

As cholinergic neurons participate in social buffering and P11 is widely expressed in the central nervous system [35] (Fig. S3A), but with enrichment in cholinergic neurons of Nac and LDT (Fig. 5A, B), we suggested a possible specific function of P11 in cholinergic neurons.

We examined the behavior of ChAT-cP11-KO (*n* = 9) and P11^flx/flx^-WT (*n* = 14) mice in the DLB without or with the introduction of a WT conspecific (Fig. 5C-E). In the DLB, we found no significant difference in the time spent in light areas in both groups (Fig. 5C). Following the introduction of a WT conspecific, we found a significantly lower time spent in the light area in ChAT-CP11-KO compared to P11^flx/flx^-WT mice (Fig. 5D) as well as time sniffing (Fig. 5E). This confirms that P11 expression in cholinergic neurons participates in social anxiety rather than general anxiety. We next tested the animals in the EDT (Fig. 5F, G). While P11^flx/flx^-WT mice discriminated between a stress and a neutral animal, ChAT-cP11-KO did not. Moreover, ChAT-cP11-KO mice even presented a reduced social interaction (Fig. 5F), an effect also visible in the discrimination index (Fig. 5G).

Several cholinergic structures could be involved in the various phenotypes related to P11 observed here [20]. To test the circuits involved in social motivation and emotion discrimination, we rescued P11-expression in ChAT-cP11-KO mice using the injection of AAV-loxP-P11-RFP [20, 43] in the basal forebrain (BF) (AAV-P11-BF, *n* = 6, Fig. 5H), LDT (AAV-P11-LDT, *n* = 6, Fig. 5I) or Nac (AAV-P11-NAC, *n* = 5, Fig. 5J) of ChAT-cP11-KO mice. We next compared the behavior with P11-WT mice (*n* = 12, injected with AAV-loxP-P11-RFP) and ChAT-cP11-KO mice (*n* = 9).

In DLB, we found a significant group effect in the time spent in the light area following re-expressing P11 in LDT and Nac, but not BF (Fig. 5K). Interestingly, the introduction of a WT-conspecific in the light area confirmed that ChAT-cP11-KO mice display a strong reduction of the time spent in the light area, and that P11-rescue in LDT, but not BF or Nac, was sufficient to prevent the effect in time spent in light area or time sniffing the stimulus mice (Fig. 5L, M). Finally, we tested the animals in the EDT test following the re-expression of P11. While P11-WT mice displayed a discrimination between the stress and neutral stimulus (Fig. 5N, O), ChAT-cP11-KO mice did not (Fig. 5F). However, the discrimination effect in ChAT-cP11-KO mice was restored with P11-rescue in BF and LDT, but not Nac, as observed by the discrimination index (Fig. 5N, O).

These findings suggest that rescuing P11 in cholinergic interneurons of Nac partially revokes anxiety, whereas P11 rescue in BF improves emotion discrimination and P11 rescue in the LDT improves both social anxiety and emotion discrimination.

### Nicotine and muscarinic cholinergic receptors modulate social anxiety and emotion discrimination differently

Our findings in ChAT-cP11-KO mice suggest that P11 expression in specific cholinergic populations may play a key role in regulating social anxiety and emotion discrimination. To investigate whether cholinergic system activation could alleviate these deficits, we administered systemically nicotine (0.15 mg/kg, i.p.), a dose previously shown to enhance social behavior in rats [44] to both P11-WT (*n* = 17) and P11-KO (*n* = 10) mice. As expected, P11-KO mice exhibited heightened basal anxiety in the DLB (Fig. S5A), increased social anxiety in the presence of a WT conspecific (Fig. S5B–C), and impaired emotion discrimination in the EDT (Fig. S5E–F). However, nicotine treatment did not produce any significant improvement in these behavioral domains in either genotype. This lack of effect may stem from nicotine’s complex action in multiple brain structures. To investigate the effects of acetylcholine in the NAc, we implanted cannulas in P11-WT (*n* = 7) and P11-KO (*n* = 6) mice to locally administer saline (0.5 µl), nicotine (100 ng/0.5 µl) [45], the muscarinic receptor agonist oxotremorine (oxo, 0.1 µg/0.5 µl) [46], or the acetylcholinesterase inhibitor tacrine (1 µg/0.5 µl) [47] (Fig. 5P). After recovery, animals were tested in the DLB. As expected, global P11-KO mice showed a significant anxiety phenotype, but none of the compounds rescued or improved anxiety in either P11-KO or P11-WT mice (Fig. 5Q). When introducing a WT conspecific, we confirmed that P11-WT mice increased the time spent in the light box under saline conditions, whereas P11-KO mice did not. Interestingly, nicotine administration was sufficient to rescue the social anxiety phenotype in P11-KO mice, without affecting P11-WT responses (Fig. 5R). In contrast, oxotremorine injection significantly reduced the time spent in the light box in P11-WT mice, while having no effect in the P11-KO mice (Fig. 5R). Tacrine injection had no significant effects compared to saline (Fig. 5R).

Finally, animals were tested in the EDT. Under saline conditions, P11-WT mice were able to discriminate between stressed and neutral conspecifics, an ability absent in P11-KO mice (Fig. 5S), as confirmed by their discrimination index (Fig. 5T). Following nicotine administration, P11-WT mice showed reduced sociability without affecting discrimination, while P11-KO mice show a trend, but yet not significant, for improvement in their ability to discriminate between stressed and neutral conspecifics (Fig. 5S, T). Oxotremorine had the opposite effect, significantly impairing social interaction and discrimination in P11-WT mice (Fig. 5S, T). Tacrine had mixed effects, reducing sociability and discrimination in P11-WT mice, while increasing sociability in P11-KO mice without significantly rescuing their emotional discrimination deficits (Fig. 5S, T).

These results suggest that acetylcholine differentially modulates social behavior through a balance of nicotinic and muscarinic signaling with only nicotine rescuing phenotypes in p11-KO mice.

### Effects of oxytocin on anxiety and social phenotype

**Fig. 6:**
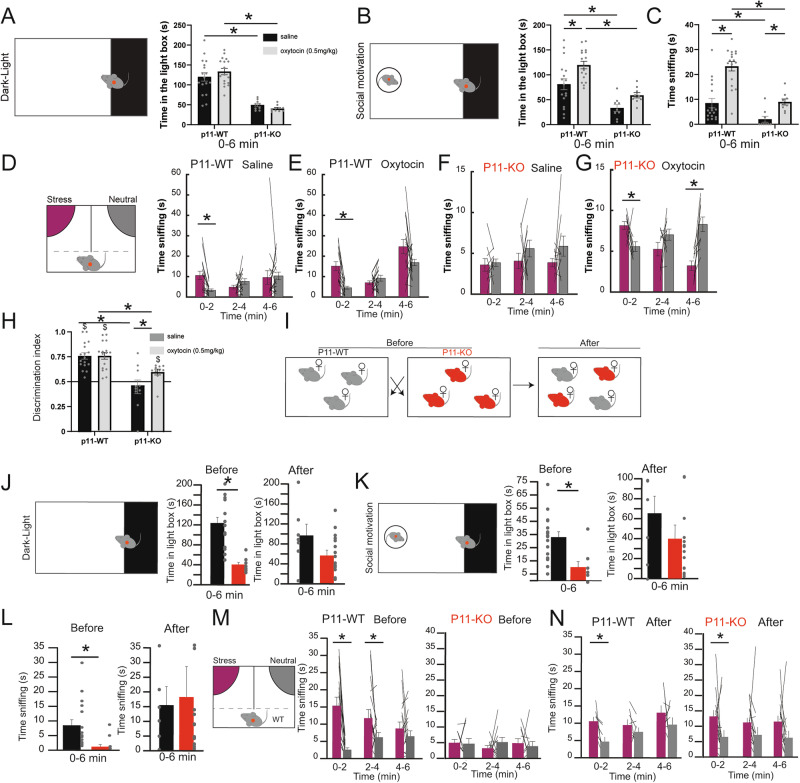
Oxytocin and social rescue specifically affect emotion-related depressive-like phenotype. (**A**) Time in the light zone during the entire dark-light box test (0-6 min) for P11-WT or P11-KO following saline or oxytocin injection (0.5 mg/kg, i.p.). 2-way RM-ANOVA group effect F(1,24) = 65.02, P < 0.0001, drug F(1,24) = 0.056, *P* = 0.81, interaction F(1,24) = 1.574, *P* = 0.2217. (**B**) Time in the light zone during the entire dark-light box test with a WT-conspecific introduced in the light area (0-6 min) for P11-WT or P11-KO following saline or oxytocin injection. 2-way RM-ANOVA group effect F(1,24) = 43.56, *P* < 0.0001, drug F(1,24) = 8.034, *P* = 0.0092, interaction F(1,24) = 0.33, *P* = 0.5679. (**C**) Time sniffing the WT stimulus during the entire dark-light box test with a WT-conspecific introduced in the light area (0-6 min) for P11-WT or P11-KO following saline or oxytocin injection. 2-way RM-ANOVA group effect F(1,24) = 23.72, *P* < 0.0001, drug effect F(1,24) = 38.48, *P* < *0.0001*, interaction F(1,24) = 4.960, *P* = 0.0356. (**D-G**) Time spent sniffing the stress (purple) or neutral (grey) stimulus in the emotional discrimination test for each segment of 2 min (0-2, 2-4, and 4-6 min) for P11-WT or P11-KO following oxytocin injection. Paired t-tests P11-WT-saline: 0-2 min *P* = 0.0004, 2-4 min *P* = 0.1156, 4-6 min *P* = 0.814; P11-WT-oxytocin: 0-2 min *P* = 1.10^-4, 2-4 min *P* = 0.1822, 4-6 min *P* = 0.0583; P11-KO-saline: 0-2 min *P* = 0.74, 2-4 min *P* = 0.30, 4-6 min *P* = 0.19, P11-KO-oxytocin: 0-2 min *P* = 0.0165, 2-4 min *P* = 0.17, 4-6 min *P* = 0.0026. (**H**) Discrimination index for EDT 2-way RM-ANOVA group effect F(1,24) = 28.71, *P* < 0.0001, drug F(1,24) = 3.647, *P* = 0.0677, interaction F(1,24) = 3.56, *P* = 0.0707. Unpaired t-test to 0.5 P11-WT saline *P* = 3.10^-8, P11-WT oxytocin *P* = 8.10^-6, P11-KO saline *P* = 0.463, P11-KO oxytocin *P* = 0.0036. (**I)** Schematic representation of the social rescue protocol between P11-WT (black) and P11-KO (red) female mice. (**J**) Time in the light zone during the entire dark-light box test (0-6 min) for P11-WT (black) and P11-KO (red) before and after social rescue. Unpaired t-tests Before *P* = 0.0001, After: *P* = 0.0502. (**K**) Time in the light zone during the entire dark-light box test with a WT-conspecific introduced in the light area (0-6 min) for P11-WT (black) and P11-KO (red) before and after social rescue. Unpaired t-tests Before *P* = 0.0072, After: *P* = 0.3388. (**L**) Time sniffing the WT stimulus during the entire dark-light box test with a WT-conspecific introduced in the light area (0-6 min) for P11-WT (black) and P11-KO (red) before and after social rescue. unpaired t-tests P11-WT versus P11-KO before: *P* = 0.029, after social mix: *P* = 0.98. (**M, N**) Time spent sniffing the stress (purple) or neutral (grey) stimulus in the emotional discrimination test for each segment of 2 min (0-2, 2-4, and 4-6 min) for P11-WT or P11-KO before (**M**) and after (**N**) social rescue. paired t-tests Before: P11-WT 0-2 min *P* = 4.10^-5, 2-4 min *P* = 0.0047, 4-6 min *P* = 0.38, 0-6 min *P* = 0.0002; P11-KO 0-2 min *P* = 0.76, 2-4 min *P* = 0.58, 4-6 min *P* = 0.86, 0-6 min *P* = 0.934, After: P11-WT: 0-2 min *P* = 0.0181, 2-4 min *P* = 0.3518, 4-6 min *P* = 0.3851, 0-6 min *P* = 0.0737; P11-KO: 0-2 min *P* = 0.0213, 2-4 min *P* = 0.1559, 4-6 min *P* = 0.083, 0-6 min *P* = 0.0603. Data are represented as mean ± SEM. Individual data are represented as dots when possible. Lines between histograms are used to represent time in a zone or interaction between conditions. *P < 0.05. $ P < 0.05 in comparison with 0.5 DI.

Since our conditional P11-KO experiments in cholinergic neurons suggest a role in social anxiety and emotion discrimination, we hypothesized that restoring social interaction might be sufficient to alleviate both social anxiety and emotion discrimination deficits. Oxytocin has been shown to modulate social anxiety and emotional processing through its pro-social effects [10, 48]. To test this, we investigated the impact of saline (1 h) or oxytocin (0.5 mg/kg i.p., 1 h) treatment in P11-WT (*n* = 17) and P11-KO (*n* = 10) mice using the DLB (Fig. 6A). As expected, P11-KO mice spent significantly less time in the light area than P11-WT mice, regardless of treatment, indicating that oxytocin did not rescue basal anxiety (Fig. 6A). We then introduced a WT conspecific in the light zone to assess social anxiety. Under saline conditions, P11-KO mice spent less time in the light zone than P11-WT controls (Fig. 6B). However, oxytocin administration significantly increased the time spent in the light area in P11WT mice, suggesting enhanced social approach behavior elicited by this compound (Fig. 6B). Additionally, oxytocin significantly increased social sniffing in both P11-WT and P11-KO mice (Fig. 6C). To further assess emotional discrimination, we tested the same animals in the EDT (Fig. 6D–G). In the saline condition, P11-WT mice successfully discriminated between neutral and stressed demonstrators (Fig. 6D), while P11-KO mice did not (Fig. 6F). Oxytocin-treated P11-WT mice were able to discriminate in the EDT, similarly, to saline-treated P11-WT mice (Fig. 6E). Importantly, oxytocin administration restored discrimination ability in P11-KO mice (Fig. 6G). This effect was further supported by analysis of the discrimination index (Fig. 6H), indicating that oxytocin treatment is sufficient to rescue social anxiety and emotion discrimination deficits in P11-KO mice.

### Social mixing improves depressive-related phenotypes

As P11-KO mice display an anxious phenotype as well as social impairment, we hypothesized that the presence of a cage mate with a normal phenotype could improve their social interaction behavior. To test if social mixing could improve anxiety and social motivation, we used female adult P11-WT (*n* = 7) and P11-KO mice (*n* = 15, Fig. 6I-N). Following initial testing, we mixed P11-WT and P11-KO mice to have an equal distribution of both genotypes in all cages for 1 month. For comparison, we examined female adult P11-KO mice mixed with other P11-KO mice (*n* = 4, Fig. S6).

In the DLB before social mixing, we confirmed that P11-KO mice spent significantly less time in the light area compared to P11-WT mice (Fig. 6J) and this difference was abolished following social mixing (Fig. 6J). Similarly, when a P11-WT conspecific was introduced in the light area, before the social mixing we observed a significant difference in the time spent in the light areas between P11-WT and P11-KO mice (Fig. 6K) an effect abolished as well following social mixing (Fig. 6K, L). These findings suggest that the presence of conspecifics that do not display an altered emotional state is anxiolytic and thus can improve social behavior in depression-like models.

We next tested the effect of social mixing in the EDT (Fig. 6M, N). Before social mixing, P11-WT, but not P11-KO, presented discrimination for a stress stimulus (Fig. 6M). In sharp contrast, following social mixing, both P11-WT and P11-KO were able to discriminate between neutral and stressed mice (Fig. 6N).

We also confirmed that mixing P11-KO mice (Fig. S6) did not improve the time spent in the light area (Fig. S6B-C). The introduction of a P11-WT conspecific in the light side did not improve the time spent in the light area (Fig. S6D-E) or in the time sniffing (Fig. S6F). Similarly, in the EDT, the P11-KO mice did not display any preference for stress mice before or after social mixing (Fig. S6G-H), suggesting that the effect observed following mixing P11-WT and P11-KO mice is related to specific emotional states rather than social mixing itself. Altogether this data suggests that the presence of cage mates presenting a normal phenotype might be sufficient to improve anxiety, social motivation, and emotion discrimination in impaired mice (Fig. S6I).

## Discussion

Our study revealed a relationship between social and emotional disabilities, commonly observed in MDD, and dysfunctions of the corticoaccumbal pathway, modulated by dopamine and acetylcholine. Using environmental and P11-based genetic models inducing depressive- and anxiety-related behavior, we found similar increases in social and general-anxiety, as well as inability to discriminate conspecific emotional states. Interestingly, findings from CSP and P11-KO mice were reminiscent of what has been observed in MDD patients [6, 49]. In direct relation to the impairment of social behavior, we found a dysfunctional synchronicity between mPFC and Nac, which coincided with an increased activity of cortical neurons as well as an increased response to excitatory inputs in Nac. Considering the role of dopamine and acetylcholine in modulating corticoaccumbal pathways at the level of the pyramidal soma and their terminals [50, 51], we found that the release of both neuromodulators during behavioral interaction with a conspecific is impaired in P11-KO mice. Our observation combining in vivo and ex vivo electrophysiology demonstrates the role of P11 in the modulation of cortical neuron firing rates as well as the synchronicity between the local field potentials of mPFC and Nac. We showed that P11 expression in specific cholinergic structures was specifically involved in basal anxiety, social motivation, and emotion discrimination. Finally, we also unraveled the possible therapeutic effect of the prosocial compound oxytocin as well as the psychotherapeutic effects of social buffering.

### Dopamine and acetylcholine balance in modulating social behavior

Dopamine release can be modulated at the level of the soma by cholinergic inputs from the brainstem [27, 52] or at their terminals in Nac by cholinergic interneurons [23]. Interestingly, acetylcholine release from LDT-originating neurons in VTA modulates motivation [27], anxiety [53], stress [30], or social reward [54]. In addition, the release of acetylcholine in Nac [55] modulates social behavior. In contrast, local dopamine release within VTA mostly reduces the activity of neighboring DA-neurons [26] while also modulating Ach release in Nac [56].

This discrepancy in social encoding of acetylcholine was further demonstrated herein by the difference in dopamine and acetylcholine balance at the level of VTA and Nac, together with the reduced neurotransmitter release and electrophysiological responses to anxious and social conditions at the cortical level in P11-KO mice. It is interesting to note that despite a common origin at VTA level, we found that the release of dopamine locally or at the terminal levels (i.e., Nac) is different, which highlights the critical role of local dopamine release in VTA [26]. This is in agreement with numerous reports showing a little-to-no relationship between dopaminergic neuron activity and dopamine release [57, 58]. Therefore, this effect underscores the possible role of synaptic modulation of dopamine release, either by cortical inputs or by cholinergic interneurons [23, 59] as well as brainstem inputs [29]. Here, we showed that specific suppression of P11 in cholinergic neurons is sufficient to impair the ability to discriminate emotion from a conspecific without impacting anxiety levels. In light of the variety of cholinergic structures [25, 28] that abundantly express P11 [35], we highlighted the individual function of the main structure using a viral gene rescue approach. Indeed, P11-rescue in cholinergic LDT neurons was sufficient to improve anxiety, social motivation, and EDT suggesting a complex function that can be linked to their projections towards VTA [27, 60] or Nac [28, 32, 60]. In comparison, P11-rescue in Nac seems to have effects restricted to anxiety, similar to previous observations [16], while rescue in forebrain seems to improve EDT. The complexity of the brainstem and forebrain cholinergic projections [27] confirm an effect of acetylcholine based on the target [27]. Our pharmacological approach, with injections of nicotine, muscarinic receptor agonist or acetylcholine esterase inhibitor suggest that not only the timely release of acetylcholine in the NAc is crucial, but also the postsynaptic targets. In the NAc, different nicotinic and muscarinic receptors are modulating dopamine and glutamate release [61–63], often through a presynaptic modulation [62] or by the modulation of cholinergic interneurons synchronization [23, 29]. Altogether, the balance between nicotine and muscarinic receptor have been shown to oppositely impact cue-motivated behavior through timely and spatially regulating dopamine release [64]. These effects become more relevant in the context of extrinsic cholinergic inputs arising from the brainstem [29, 32, 59] as well as an hyperactive cortex in P11-KO mice. Interestingly, both nicotinic and muscarinic receptors co-exist within the VTA to modulate anxiety [42, 65] and social stress responses [45, 66]. However, the effects of acetylcholine in the NAc appear to be more specifically related to social and emotional processing than those observed in the VTA. In particular, we found that nicotine counteract the deficits in social anxiety and emotional discrimination in P11-KO mice Altogether, these observations highlight the role of acetylcholine and nicotine in social behaviors [67] and the modulation of the corticoaccumbal pathway [68, 69].

### Pro-social and social-rescue strategies

Lack of social motivation is frequently comorbid with MDD, and in some cases precedes other symptoms [70]. PTSD and autism are also characterized by reduced social motivation [71, 72] as well as emotion discrimination. It is well-established that oxytocin plays a crucial role in social bonding, trust and empathy through complex neural circuits [10, 73]. Of interest to our study, intranasal oxytocin shows promise as a treatment for various mental health conditions [73] including MDD and anxiety [74, 75]. In particular,oxytocin agonists appear to alleviate depressive symptoms [76]. Interestingly, we found that the administration of oxytocin, but not nicotine, was sufficient to counteract social-related anxiety while improving emotion discrimination, but with limited effects on general anxiety, in both P11-WT and P11-KO mice. These findings further suggest that the mechanisms encoding basal anxiety and social anxiety are differently encoded by neural circuitry.

Beyond pharmacological treatment, behavioral psychological therapies are crucial for the management of various forms of anxiety, including social motivation, as well as MDD [77]. One major aspect of psychotherapy in treating these psychiatric manifestations is focusing on counteracting social isolation [78, 79]. This therapy is known as a social rescue and has been shown to cause long-lasting improvement against MDD [80]. In our social-rescue protocol, cage shuffling between mice with different genotypes and anxiety levels, not only improves social motivation but also emotion discrimination, without affecting the basal anxiety. These improvements were not found within mice with the same anxiety level. Our findings highlight the crucial role of social interaction in counteracting anxiety and emotion discrimination impairments observed in MDD models.

### Technical limitations and conclusion

Our study is the first to uncover the role of P11 in social anxiety and emotional discrimination, two behavioral domains critically implicated in MDD. While we employed a range of state-of-the-art methods to investigate the contribution of P11 in specific neuronal populations, including behavioral, in vivo and ex vivo electrophysiology, and pharmacological approaches, several technical limitations should be acknowledged.

First, our ability to temporally dissect the function of the cortico-accumbal pathway was constrained. Chemogenetic tools, while cell-type specific, lack the temporal resolution required to synchronize activity between the cortex and NAC, and therefore could not be used to fully re-establish functional circuit dynamics. Similarly, optogenetic approaches, though temporally precise, were technically limited in our setup, particularly in socially interactive contexts or in freely moving animals housed in their home cages. The synchronization of optogenetic stimulation with endogenous cortical activity and terminal activation in the NAC proved technically challenging under these conditions.

To probe the functional relevance of the cortico-accumbal pathway, we used two independent strategies to increase social interaction in P11-KO mice: a pharmacological approach using oxytocin and a behavioral “social rescue” paradigm. Both interventions were sufficient to restore social behavior and emotion discrimination but failed to reverse elevated basal anxiety. However, these approaches lack the specificity required to identify the contribution of particular neuronal subtypes or neurotransmitters. These approaches with rescuing social interaction, while crucial, require additional work to specifically dissect the neural circuits involved in the rescue of such phenotypes.

Altogether, we provide evidence for a novel role of the balance between dopamine and acetylcholine in the modulation of cortical-striatal activity, and in the ability to control social interaction and emotion discrimination in both environmentally and genetically induced depression-like phenotypes. In addition, our data supports a role for oxytocin and, intriguingly, also social rescue, to alleviate anxiety, and improve social motivation as well as emotion discrimination in mice with socially linked depression-/anxiety-like states.

## Supplementary information

Supplementum

Supplementary Table

## Data Availability

All data are available in the main text or the supplementary materials.
