# Supplementary material for: Altered acetylcholine modulations and corticoaccumbal pathway in P11-linked social dysfunction
Source: Mol Psychiatry. 2025 Nov 3;31(3):1785–98. doi: 10.1038/s41380-025-03324-2 (PMC12916318; doi:10.1038/s41380-025-03324-2)
Supplement: Supplementary file 1 — Supplementum [file 41380_2025_3324_MOESM1_ESM.docx]

Supplementary Information

**Altered acetylcholine modulations and corticoaccumbal pathway in p11-linked social dysfunction.**

Dautan Daniel Ph.D^1*^, Anderson Camargo Ph.D ^1^, Niclas Branzell^1^, Valentina I. Brioschi^1^, Daniel Doyon^1^, Elisa Covatta^1^, Roberta Marongiu Ph.D ^3^, Michael Kaplitt MD. Ph.D ^3^, Karima Chergui Ph.D ^2^, Xiaoqun Zhang MD, Ph.D ^1^, Per Svenningsson MD. Ph.D ^1*^

1. Department of Clinical Neuroscience, Karolinska Institute, Stockholm, Sweden

2. Department of Physiology and Pharmacology, Karolinska Institute, Stockholm, Sweden

3. Department of Neurosurgery, New-York Hospital-Cornell Medical College, New York, NY, USA.

* Corresponding authors: Daniel Dautan, daniel.dautan@ki.se; Per Svenningsson, per.svenningsson@ki.se

**Materials and Methods**

**Animals**

Adult Wild-Type (WT, C57Bl6, 8-12 weeks), P11-WT, constitutive P11-knockout (P11-KO), constitutive P11-knockdown (P11-HET) or conditional P11 knockout in choline acetyltransferase (ChAT-cP11-KO) and their respective controls (P11^flx/ flx^) were generated on-site as previously described (1,2). All animals were maintained in house cages with cage mates of similar genetic backgrounds (WT, P11-KO, P11-HET, and P11-WT) or mixed (ChAT-cKO-P11, P11^flx/ flx^). Animals had ad libitum access to food and water across all experiments and were maintained on a strict 12h light-dark cycle. Both males and females were used, and their genetic background was confirmed by PCR. All experiments were conducted and approved by the Karolinska Institute Ethical Committee (3218-2022) according to the Swedish guidelines and conducted in accordance with the European Communities Council Directive of 24 November 1986 (86/609/EEC). They were housed in temperature- and humidity-controlled rooms (20°C, 53% humidity) with a 12 h dark/light cycle. They had access to standard lab pellets and water *ad libitum*.

**Chronic restraint stress protocol**

Adult WT mice (male and female) were first handled for 2 consecutive days prior to further experiments. Next, the animals were divided into 3 groups; mice that were handled 2 minutes daily for 24 days (CTRL), mice that were handled 2 minutes daily for 14 days followed by 10 days of chronic restrain protocol (CSP), and mice that directly underwent the chronic restrain protocol for 10 days, before being handled for the remaining 14 days (CSP+2w). Chronic restrain protocol was repeated daily at a regular schedule for each animal. At the beginning of the sessions, the animals were gently moved to an experimental room and let to acclimate for 15 minutes. Next, animals were gently transferred to a 50ml Eppendorf tube with the end cut off to allow breathing. Tubes were then fixed horizontally for 2h before releasing the mice into their home cage. Finally, mice were let to acclimate for 15 additional minutes prior to being returned to their room.

**Systemic drug experiments**

Saline, oxytocin (#1910, Tocris) and nicotine (nicotine tartrate salt, sml1236-50mg, Merck) were used for the experiments and prepared freshly. Oxytocin was injected i.p at 0.5mg/kg and tested 1h after administration, nicotine was used at 0.15mg/kg and tested 30min after administration. Both drugs were diluted in saline and the same volume was administered as a control.

**Immunohistochemistry**

After *in vivo* experimental procedures, the animals were deeply anesthetized with an overdose of sodium pentobarbital (200mg/kg) and transcardially perfused with 20ml of ice-cold phosphate buffer solution (PBS, 0.05M) followed by 20ml of fresh paraformaldehyde (PFA 4%). The entire brain was then removed, post-fixed in PFA for 4-8h, and then transferred in a cryo-protection solution (30% sucrose in PBS). Following 2-3 days of cryoprotection, brains were embedded in a mounting medium for cryostat (OCT, Fisher scientist) and sliced coronally at 25µm thickness. Immediately following slicing, sections were transferred to a cryoprotective solution and kept at -20°C until staining. Before staining, sections were transferred to well plates containing PBS at room temperature. All immunohistochemistry solutions were prepared in a solution of PBS with 0.3% Triton (PBS-Triton). First, sections were blocked in PBS-Triton containing 10% normal donkey serum (NDS, Jackson Immunoresearch) for 1h at room temperature. Following 3-5 washes, sections were incubated in the solution containing PBS-Triton, 1% NDS, and the corresponding primary antibody overnight at 4 °C under constant and gentle shaking. The following day, sections were washed 3-5 times with PBS and thus incubated in the solution containing PBS-Triton, 1% NDS, and the corresponding secondary antibody for 4-5h at room temperature under constant and gentle shaking. Sections were then washed 3-5 times in PBS before being mounted on microscope slides using a mounting medium. Primary antibodies were as follows: mCherry (raised in mouse, concentration 1:1000; ABCAM AB167477), GFP (to enhance eYFP staining, made in Rabbit, already conjugated to Alexa-488, concentration 1:1000, Thermofisher, A21311) DAT (dopamine acetyltransferase, made in rabbit, concentration 1:1000; Millipore Sigma, MAB369), P11 (S100A10, raised in mouse, concentration 1:200; Cell signaling, 5529s) and DAPI (Sigma-Aldrich, D9542-5Mg). The secondary antibody was as follows Alexa-488 against rabbit (Jackson Immunoresearch 611-545-215), Alexa-488 against mouse (Jackson Immunoresearch 615-005-214), and Alexa CY3 against mouse (Jackson Immunoresearch 615-165-214).

All sections were thus scanned using a fluorescent confocal microscope (Carl Zeiss LSM 880) using high-resolution (2048*2048) images at 10X or 20X magnification. For single neuronal quantification, images were acquired using a 63X in-oil objective. Z-stack and tile-scan were acquired at reasonable parameters for each slice. Further analyses and modifications of images were done using Fiji software (ImageJ). Cell counting was done using the in-build multi-point tools and normalized by the surface scan. Fluorescence for specific areas was done using DAPI staining to delimitate the area and measure the fluorescence using the in-build measure tool. For each animal, 3 tiles scanned were used at similar levels across the entire brain structure. For single-cell fluorescence quantification, a same-size square centered on each neuron was used and the signal to the background was used to normalize the soma-fluorescence intensity.

***In situ* hybridization**

The whole brain from WT, P11-WT, and P11-KO were collected and snap-frozen in isopentane, cooled in dry ice, and subsequently stored at -80ºc. Coronal sections (12µm thick) were collected at the level of the dorsal raphe nucleus (DRN), the Laterodorsal Tegmental nucleus (LDTg), the nucleus Accumbens (NAC), and the medial prefrontal cortex (mPFC) using a cryostat section (Leica, Kista, Sweden) and mounted on Polysine glass slides (VWR, Stockholm, Sweden). Sections were fixed in 4% Paraformaldehyde and further processed. A 35S-labeled anti-sense cRNA probe against P11 was prepared by in vitro transcription from a cDNA clone corresponding to the S100a10 gene. The transcription was performed from 100 ng of linearized plasmid using 35S-UTP (1000 Ci/mmol) and T3 RNA polymerase. In situ hybridization was performed as previously described (4). Briefly, 12 μm thick cryostat sections were postfixed in 4% PFA for 5 min at room temperature, rinsed twice in 4 × sodium chloride–sodium citrate buffer (SSC), and placed into 0.25% acetic anhydride in 0.1 M triethanolamine/4 × SSC (pH 8) for 10 min at room temperature. After dehydration in graded alcohols, the sections were hybridized overnight at 55°C with 35S-labeled P11 probe in 50 μl of hybridization solution (20 mM Tris–HCl/1 mM EDTA/300 mM NaCl/50% formamide/10% dextran sulfate/1 × Denhardt’s/250 μg/ml yeast tRNA/100 μg/ml salmon sperm DNA/0.1% SDS/0.1% sodium thiosulphate). The slides were washed in 4 × SSC (5 min, four times), RNAse A (20 μg/ml) (20 min, at 37°C), 2 × SSC (5 min, twice), 1 × SSC (5 min), 0.5 × SSC (5 min, twice) at room temperature, and rinsed in 0.1 × SSC at 65°C (30 min, twice) (all washes contained 1 mM DTT), before being dehydrated in graded alcohols. The slides were then exposed to X-ray films for 10 days. Autoradiograms were digitized using a Dia-Scanner (Epson Perfection 4870 PHOTO). Optical density values were measured using Image J (1.52 h, Wayne Rasband National Institutes of Health, United States).

**RNAScope**

For fluorescent ISH (RNAscope), section staining was performed using the RNAscope Multiplex Fluorescent assay (Advanced cell diagnostics, Abingdon, Oxford). Fresh frozen sections were post-fixed in 4% PFC for 15 min at 4°c and dehydrated in graded alcohols. Afterward, a protease IV (Advanced cell diagnostics) was applied for 30min at room temperature. Afterward, sections were hybridized with the probes for P11 (Mn-S100A10, cat. 410901) for 2h at 40ºc. The hybridization step was followed by standardized steps of amplification (Amp 1-FL 30 min at 40 °C, Amp 2-FL 15 min at 40 °C, Amp 3-FL 30 min at 40 °C, Amp 4C-FL 15 min at 40 °C). After the last amplification step, sections containing cholinergic structures were counterstained with polyclonal ChAT primary antibody (AB1582, Abcam) and an anti-goat Alexa-Fluor 568 secondary (Invitrogen). Sections were mounted with Dako fluorescent mounting medium and imaged on Carl Zeiss LSM 880 confocal microscope using either 20x objective or a 63x in oil immersion objective. Z-stacks of 7-10µm thickness were obtained in each caption.

**Autoradiography**

Fresh frozen sections mounted on Superfrost slides were used for autoradiographic detection of DAT. Sections were pre-incubated in binding buffer (50 mM Tris–HCl/120 mM NaCl, pH 7.5) for 20 min. They were incubated in binding buffer with 50 pM [^125^I] RTI-55 (Perkin-Elmer Life Sciences) and 1 μM fluoxetine (Tocris Bioscience) for 60 min. For non-specific binding, 100 μM nomifensine (Sigma-Aldrich) was added to the assay. Lastly, sections were washed in ice-cold binding buffer for 2 × 10 s and rapidly dipped in deionized water. When dried, sections were exposed to Kodak Biomax MR Film (Sigma-Aldrich) in a dark room. After 24 h, autoradiograms were digitized using a high-resolution scanner (Epson Perfection V750 PRO). Optical densitometry was done with Image J in grayscale in the same way as aforementioned.

**Western blot**

After treatment, the mice were sacrificed, and the brains were removed and placed in PBS. Brains were then carefully desiccated to extract the mPFC, the nucleus accumbens and the ventral midbrain containing the ventral tegmental area. Immediately after extraction, tissue was frozen and kept at -80 °c until further manipulation. Tissues were homogenized using sonication and proteins were extracted using RIPA buffer (Pierce, #89901). Protein concentrations of the lysates were determined using (BCA protein assay kit) following the manufacturer’s instructions, and volumes of lysates were mixed with appropriate volumes of 4x Laemmli sample buffer (Biorad, #1610747) such that all samples had the same protein concentration. Equal volumes of samples (10µL) were pipetted into each lane of a NuPAGE 4-12% Bis-Tris precast gels (Invitrogen, NP0323BOX) and separated at 120V, before transferring to PVDF membranes (Biorad, 1704275) using the Trans-blot turbo transfer system (Biorad) as per the manufacturer’s instructions. Membranes were blocked for 2 hours at room temperature in 5% skim milk powder (Sigma, ref 70166) in TBST buffer under constant shaking. Membranes were then incubated overnight at 4°C with the following antibodies: PSD-95 (Pierce, MA1-045, 1:1000, mouse) or GAPDH (Santa Cruz, sc-32233*,*1:1000, mouse) under constant shaking. The membranes were washed three times in TBST before incubation with the autofluorescent secondary antibody (Li-cor, #926-68070, 1:10 000, anti-mouse) for 2 hours at room temperature with shaking. The membranes were washed with TBST before imaging in a Chemidoc MP imaging system (Biorad). Images were processed using (*imageJ*) and fluorescence values were normalized to GAPDH signal in the same sample.

**Behavioral tests**

**Dark/lightbox:** The dark/light box was conducted in a commercially available apparatus as previously described (4). The time over 2-minute epoch windows or the cumulative time (6 minutes) of the test was manually scored by the experimenter blind to the manipulation. In short, mice were placed on the dark side (<2 lux) and given access to the light side (30-50 lux) for 6 minutes. At the end of the 6 minutes, an object (heavy dark bottle 250ml), food (45mg sugar pellets that were given to the mice for 3 days prior to testing, test diet), or a cup (5cm diameter, transparent plexiglass with holes) containing WT mice matched by age and sex were placed at the opposite side of the transition door in the light area and recording restarted for 6 minutes. For all recordings, time in the light area per 2-minute epoch or the 6 minutes of the task, as well as sniffing (object, food, or stimulus mice) were collected and analyzed. During testing, if a mouse did not enter the light area for the entire recording (6 minutes) the data were discarded and not further analyzed.

**Emotion discrimination task:** Testing mice (observers) were handled for 2 consecutive days and habituated (2 consecutive days, 10 min) inside a custom-made 3 chambers box divided with a dark separator (5). On the day of the experiments, the observer mouse was placed in the box for 6 minutes for acclimatization, then 2 stimulus mice were placed in a 5cm radius plexiglass cup with holes drilled to allow contact. Both cups were in either side of the 3-chambers with no direct reciprocal view between demonstrators. Immediately following the placement of the 2 stimulus mice, the recording was started for 6 minutes. Testing was first done with two naïve WT mice (Neutral versus Neutral), matched by sex and age to the observer) and used to determine any possible bias (habituation) at the end of the recording both stimulus and cups were replaced with 2 fresh stimuli that either presented a neutral state (WT mice), a stress state (WT mice that underwent movement restriction in a 50ml Falcon tube for 15min prior to the test) or P11-WT (test). The cups were replaced after each subject with clean copies to avoid scent carryover. The side for each stimulus was randomly assigned across experiments. The chambers were wiped with 75% ethanol and allowed to air dry between tests. During both habituation and behavioral testing, the apparatus was placed in a dim light condition (~10lux). Habituation and test videos were collected using a high-resolution camera located above the apparatus, connected to Ethovision XT tracking software (Noldus). The videos were then scored offline by experimenter’s blind to the manipulations and scoring for the time in each zone (defined as a 10*10cm around the cup of each stimulus) or the time sniffing (time were the demonstrator-initiated sniffing toward a stimulus or the cup) were extracted using custom keyboard software. Scoring for the time in zone or time sniffing was extracted and expressed per 2 minutes epoch windows or the entire recordings (6 minutes).

**Cannulation of the Nucleus Accumbens (NAc)**
P11-WT and P11-KO mice were anesthetized using isoflurane (1.5–4% in oxygen) and placed in a stereotaxic frame (Kopf Instruments) equipped with a heating pad to maintain body temperature throughout the surgery. After confirming the absence of reflexes, the scalp was disinfected with iodine solution and a midline incision was made to expose the skull. The skull was leveled by aligning bregma and lambda in the dorsoventral plane. Two small craniotomies were performed using a microdrill: the first, located above the cerebellum, allowed insertion of a stainless steel surgical screw (Agnthos) to anchor the implant, and the second was positioned above the right nucleus accumbens (NAc) based on the following stereotaxic coordinates relative to bregma: anteroposterior (AP) +1.0 mm, mediolateral (ML) +1.0 mm. A 26-gauge stainless steel guide cannula (Plastics One), 5 mm in length, was then lowered to a depth of 4.0 mm dorsoventral (DV) from the skull surface, positioning the tip just above the NAc. The cannula was secured to the skull using dental acrylic anchored to the surgical screw. Mice were returned to their home cage and monitored until full recovery. Postoperative care included administration of analgesics and daily health checks.

**Intracranial Infusions and Behavioral Testing**
Approximately 30 minutes prior to each behavioral session, animals received an intracranial infusion via an internal injector extending 0.5 mm beyond the guide cannula tip. Each infusion delivered 0.5 µl of solution over 1 minute using a microinfusion pump connected to a Hamilton syringe. The infused compounds were: sterile saline (vehicle), nicotine (nicotine tartrate, 100 ng in 0.5 µl; Sigma-Aldrich), oxotremorine (0.1 µg in 0.5 µl; Tocris), and tacrine (9-amino-1,2,3,4-tetrahydroacridine hydrochloride hydrate, 1 µg in 0.5 µl; Sigma-Aldrich). After infusion, the injector remained in place for an additional 60 seconds to allow diffusion and prevent backflow. Mice were then returned to their home cage for a 30-minute pre-testing interval. Drug administration followed a within-subject design with at least 24 hours between sessions to allow for washout of previous compounds. Behavioral testing was conducted under identical conditions across all treatment sessions.

**Stereotaxic surgery**

All surgeries were performed under aseptic conditions. Mice were deeply anesthetized with isoflurane (1.5 to 4% in O_2_) and placed in a stereotaxic apparatus (Kopf Instruments). Ophthalmic ointment was applied. Following shaving and skin incision, a small cranial hole was made above the targeted structure. All coordinates were obtained relative to the bregma and dorsoventral coordinates were from the brain surface. Viral injections were performed using a nanos-syringe (#7001, Hamilton Syringes) at 10-25nl/min using a micro syringe pump (micro4, WPI). After completion of the injection, the syringe was maintained in position for 10 to 15 min prior to withdrawing to reduce backfilling. At the end of the surgery, animals were given injections of Buprenorphine (0.1mg/kg, sc) and Baytril (0.05mg/kg, i.p). Viruses used for all experiments are as follows: Dopamine sensor (AAV9-hSyn-GRAB-rDA1h, 140557-AAV9, Addgene), Acetylcholine sensor (AAV1-CAG-iAChSnFR, 137955-AAV1, Addgene) and AAV1-2.CBA.RFP.loxP-P11 (gift from Kaplitt lab (6). Neurotransmitter sensors were mixed (1:1 ratio) and injected in the SNc (Coordinates AP: -3.5mm, ML: +/-1.5mm, DV:4.0mm, 300nl mix) or Nac (Coordinates AP: +1mm, ML: +/-1.0mm, DV:4.5mm, 600nl mix). AAV-P11-mCherry was injected in the LDT (Coordinates AP: -5.0mm, ML: 0.3mm, DV:3.3mm, 250nl bilateral), the basal forebrain (Coordinates AP: +1.0mm, ML: 0.1mm, DV:4.5mm, 300nl bilateral) or Nac (Coordinates AP: -+1.0mm, ML: +/-1.0mm, DV:4.4mm, 500nl bilateral). At the end of behavioral experiments, all injections were confirmed posthoc using immunostaining for mCherry, and animals with misplaced injections were discarded.

**Fiber photometry**

All fiber photometry experiments include tripled wavelength photometry recordings. 4 to 5 weeks following the injection of neurotransmitter sensors, a second surgery was done. Following anesthesia and preparation of the animal skull, custom-made 400µm optic fiber implants (MM, Thorlabs) connected to zirconia 1.25mm ferrules (Thorlabs) were implanted in the SNc (200µm above injection site), Nac (200µm above injection site) or the mPFC (100µm above injection site). Ferrules were maintained in position using 2-to-3 anchor screws (Agnthos) and dental acrylic cement (Agnthos). Mice were given 1 week for recovery. All experiments were done on a custom-made dark/light box comprised of a dark side (7.5*20cm, <2 lux) and a light side (25*20cm, >50lux) with a central door paired with an infra-red beam to detect entries and exit and positioned 3cm above the floor. Infra-red beam signal was collected using an Arduino board (Arduino Uno) and converted into TTL signal to be transmitted to the fiber photometry acquisition (Doric lenses). Photometry signal was collected using a 3-wavelength excitation (isosbestic 415nm, GCamP 460nm and RCAMP 540nnm) and signal collection (isosbestic 415nm, GCAMP 500nm, RCAMP 680nm) fluorescence minicube (Doric lenses) with build-in LED and Detector (ilFMC6-G2, Doric lenses) connected to each ferrule with a 400µm low autofluorescence multimode patch cord (2.5m long, Doric lenses) and to the Fiber photometry console (FPC, Doric lenses). Build-in LEDs were controlled using an LED driver (LEDD_4, Doric lenses) to control the far blue (208Hz), green (166Hz), and red (333Hz) excitation. Signal was acquired in Lock In mode and deconvolved directly onto the software. The Signal was filtered at order 12^th^. Signals were individually collected and annotated accordingly. Excitation wavelengths were adjusted to produce the best signal-to-noise ratio, with a maximum power of 50µW. Analog input signals from the infrared beam sensor and the manual scoring were collected as additional input channels.

Following habituation, mice were connected to two ferrules (VTA or Nac together with a dummy one) and given 5 minutes of recovery in their home cage prior to being transferred into the dark light apparatus. Individual recordings of 6 minutes were collected in the following order: 1) Dark light with light OFF, 2) Dark light with Light ON (>1000 lux), 3) Unfamiliar object placed in the light side light ON, 4) Familiar sugar pellets placed in the light side light ON, 5) Matched by age and sex WT mice placed under the cup in the light side light ON, 6) Matched by age and sex P11-KO mice placed under the cup in the light side light ON, 7) Matched by age and sex naïve WT mice placed under the cup with the light OFF and 8) Matched by age and sex naïve P11-KO mice placed under the cup with the light OFF. Between each animal, the apparatus and the cups were thoroughly cleaned with 75% ethanol solution.

**Fiber photometry analyses**

Raw deconvoluted signal for each wavelength (isosbestic, AChSnFr, rGRAB-DA) and sites (VTA, Nac) were extracted using an adjusted version of the Matlab script available on the Doric lenses website. In short, all entries defined by the analog inputs corresponding to the infrared beam were converted into data sampling frequency and then divided into entries and exits, further characterized as “entries to the light area” and “exit the light area”. The inter entry/exit intervals were then determined for each exploration of the light area and the dark area, and any period that lasted less than 2.5s was discarded to avoid overlapping of signal as well as oversampling. Next, each entry/exit that lasts more than 2.5s was extracted based on a 5s-epoch center on the infrared beam signal. Each window was then detrended using a polyfit function of order 1 (order 1 was defined as photobleaching on a 5s window is negligible) and normalized using z-score function. Movement artifacts on the isosbestic channel were considered to be present if the signal increase of more than ±2SD for more than 250ms, and if so, the entire entry was discarded for all channels including the AChSnFr and the rGRAB-DA signal of the same fiber. The average signal during the baseline (2.5s) and the entry period (2.5s) was thus compared using paired analyses (RM-ANOVA). For comparison between conditions (P11WT, P11-KO) the analyses focused on comparing the signal during the baseline and during entry using unpaired analyses (factorial ANOVA). No further normalization was done, and all signals were analyzed similarly.

***In vivo* Electrophysiology recording**

Mice underwent stereotaxic implantation of a subcutaneous 2-channels LFP telemetry device (F20-EET, DSI, St. Paul, MN). Before the surgery, one wire of Channel 1 and 2 was selected and soldered to a 0.1mm coated copper wire. Solder joints were then covered with epoxy and heat-shrink tubing to isolate them from noise. Copper wires were then inserted in a fused silica tubing (DigiKey), fixed in position using epoxy, and exposed for 200-300µm. The remaining wire was next soldered to an anchor screw (Agnthos, MC1X2). On the day of the surgery, mice were prepared as described above and 4 holes were drilled, 2 above the cerebellum (coordinates: AP: +2.0, ML: +1.0 from Lambda, bilateral), one above the mPFC (coordinates: AP: +1.9, ML: +0.4 from Bregma) and one above the nucleus accumbens (coordinates: AP: +1.0, ML: +1.0 from Bregma). The telemetry implant was then inserted in the pocket located on the back of the animal. The two anchor screws were then carefully inserted above the cerebellum (0.33mm from the skull surface) while the LFP electrodes were inserted in the mPFC (1.9mm from the skull surface) or Nac (4.3mm from the skull surface). Both electrodes and screws were then maintained in position using dental acrylic. Animals were then placed into their home cage and given 5 days to recover. On the day of the recording, the cage of the animal was placed on the telemetry receiver (DSI), and the implant was activated using a strong magnet. For all experiments, recordings were done in the home cage of the mice to avoid additional stress effects. Signal from the mPFC and Nac were then collected at 1000Hz using Dataquest 6.0 software (DSI) on continuous mode. Mice were first placed in a dark environment for 5 minutes (Dark), then the light was turn ON for 5 additional minutes (Light), and finally, a WT unfamiliar conspecific mice, age and sex paired was placed in the cage for 5 additional minutes. Animals were thus divided into 2 groups based on their mPFC signal. The first group presented the presence of action potential in the LFP and was used only for analyses of firing rate. The second group does not display any spike in the signal and was used for LFP analyses. No animals present spikes in the LFP recorded within Nac. Animals that present movement artifacts in their signal were not further analyzed.

***In vivo* Electrophysiology analyses**

Signal arising from animals that present action potential in the LFP signal collected within the mPFC was first filtered (band-pass filtering 20-150Hz) using function on the Neuroscore (DSI) software. Due to the presence of multiple units in each recording, a pseudo-typical spike sorting was done using the amplitude and the action potential waveform using a specific software function. Any action potential with an amplitude <2 times the noise signal was not further analyzed. No further clustering was done, and thus selected units were only considered as putative single units. Time stamps of action potential for all putative single units were then extracted and the mean firing rate (the number of action potential divided by 15 minutes), and the instantaneous firing rate (mean interspace interval) were then calculated. The instantaneous firing rate for each 200ms epoch window was then normalized using the z-score function or average for each 5min time of the behavioral task.

Signals arising from animals with no spike contamination of the mPFC LFP were first divided into mPFC or Nac signals, individually filtered (hamming, FFT) and the periodogram normalized using z-score function. In parallel, individual LFP-filtered signals were extracted and converted for analyses using Matlab custom script. The correlation index between mPFC-LFP and Nac-LFP was measured over a 30s epoch window using corrcoeff function with mPFC-LFP leading the lag signal (correlation signal mPFC towards Nac). The correlation index was then extracted for the 15 minutes of the behavioral tasks or averaged for the 5 minutes of each behavioral task.

**Slice Electrophysiology recordings**

Adult P11-WT and P11-KO mice underwent cervical dislocation and decapitation. Their brains were immediately removed, and coronal brain slices (200 μm thick) of nucleus accumbens level were prepared with a microslicer (VT 1000S; Leica Microsystem, Heppenheim, Germany). Slices were incubated in oxygenated (95% O2 + 5% CO2) artificial cerebrospinal fluid (aCSF) (126 NaCl, 2.5 KCl, 1.2 NaH_2_PO_4_, 1.3 MgCl_2_, 2.4 CaCl_2_, 10 glucose, and 26 NaHCO3, in mmol/L, pH 7.4), for 1 h at 32°C. Slices were moved to a recording chamber and were continuously supplied with oxygenated aCSF at 28°C. Extracellular field potentials were recorded using a glass micropipette filled with aCSF positioned on the surface of the nucleus accumbens. Synaptic excitatory postsynaptic potentials/population spikes (fEPSP/PSs) were evoked by stimulation pulses delivered every 15 s to the nucleus accumbens by a concentric bipolar stimulating electrode (FHC, Bowdoinham, ME, United States) placed near the recording electrode. Single stimuli (0.1 ms duration) were applied at 0–100 μA. Signals were amplified 500 or 1000 times via an Axopatch 200B or a GeneClamp 500B amplifier (Axon Instruments), acquired at 10 kHz, and filtered at 2 kHz. Data were acquired and analyzed with the pClamp 11 software (Axon Instruments, Foster City, CA, United States).

**Statistical analyses and data sharing**

All data are represented as mean±SEM. No power analyses were conducted before the experiments, and group size were determined before experiments based on comparable previously published experiments. For behavior analyses, the sides of the stress, P11-KO or neutral stimulus were randomized. Before analysis, normal Gaussian distribution was assessed using Shapiro-Wilk test. Appropriate parametric test (two-tailed unpaired or paired Student’s t-tests) was chosen best on the population distribution. 1-way ANOVA was used when comparing one variable of several population samples. 2 or 3-way ANOVA was used when analyses accounted for 2 or more distinct variables. Post-hoc Bonferroni analysis results are mentioned in each figure using *. Due to multiple comparisons, not all effects are represented in the figures, data can be found in the statistical summary. No outliers were discarded except precise in the statistical summary. All analyses were done on Statistica (TIBCO) software, and the level of significance was set at P<0.05. For behavioral experiments as well as electrophysiology/photometry, raw data, and statistical outcomes are provided in the extended statistical file. The test used, the number of samples, and the P-values can be found in the results section or the supplementary statistical file.

**Supplementary References**

1. Svenningsson P, Chergui K, Rachleff I, Flajolet M, Zhang X, El Yacoubi M, et al. Alterations in 5-HT1B receptor function by p11 in depression-like states. Science 2006;**311**:77–80.

2. Schintu N, Zhang X, Alvarsson A, Marongiu R, Kaplitt MG, Greengard P, et al. p11 modulates L-DOPA therapeutic effects and dyskinesia via distinct cell types in experimental Parkinsonism. Proc Natl Acad Sci U S A. 2016;**113**:1429–1434.

3. Heifets BD, Salgado JS, Taylor MD, Hoerbelt P, Cardozo Pinto DF, Steinberg EE, et al. Distinct neural mechanisms for the prosocial and rewarding properties of MDMA. Sci Transl Med. 2019;**11**.

4. Sousa VC, Mantas I, Stroth N, Hager T, Pereira M, Jiang H, et al. P11 deficiency increases stress reactivity along with HPA axis and autonomic hyperresponsiveness. Mol Psychiatry 2021; **26**:3253–3265.

5. Scheggia D, Managò F, Maltese F, Bruni S, Nigro M, Dautan D, et al. Somatostatin interneurons in the prefrontal cortex control affective state discrimination in mice. Nat Neurosci. 2020; **23**:47–60.

6. Alexander B, Warner-Schmidt J, Eriksson TM, Tamminga C, Arango-Lievano M, Ghose S, et al. Reversal of Depressed Behaviors in Mice by p11 Gene Therapy in the Nucleus Accumbens. Sci Transl Med. 2010;**2**:54–76.


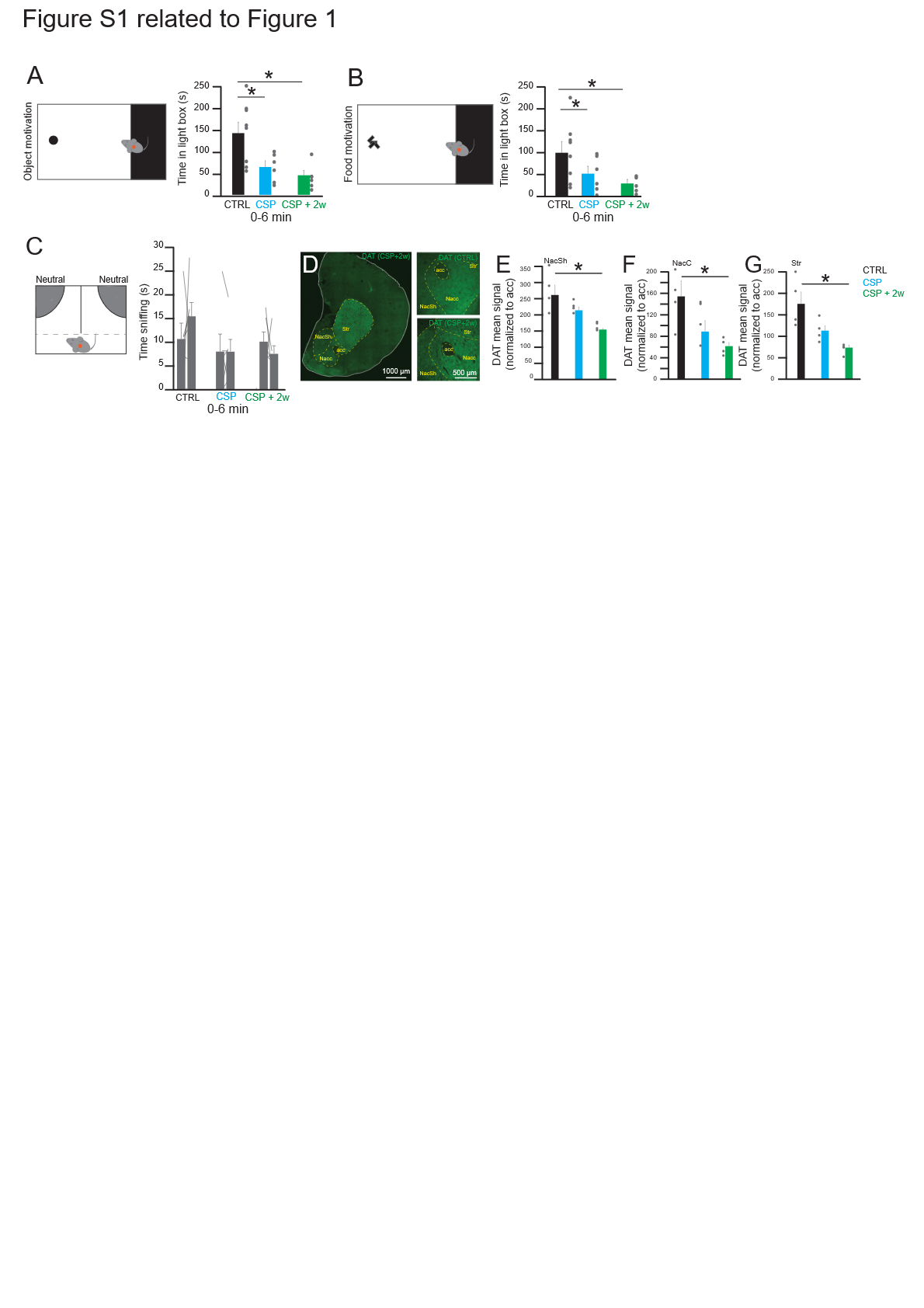


(**A**) Time in the light zone during the entire dark-light box test with an unfamiliar object introduced in the light area (0-6 min) for CTRL (black), CSP (blue), and CSP+2w (green). 1-way ANOVA F(2,17)=7.13, *P=*0.0056, post hoc CTRL versus CSP *P=*0.036, CTRL versus CSP+2w *P=*0.0084. (**B**) Time in the light zone during the entire dark-light box test with familiar sugar pellets introduced in the light area (0-6 min) for CTRL (black), CSP (blue), and CSP+2w (green). 1-way ANOVA F(2,17)=7.81, *P=*0.0039; post hoc CTRL versus CSP *P=*0.031, CTRL versus CSP+2w *P=*0.00551. (**C**) Time spent sniffing neutral (grey) stimuli during the entire habituation of the emotional discrimination test (0-6 min) for CTRL (black), CSP (blue), and CSP+2w (green). paired t-tests, *P>*0.05. (**D**) Confocal images of dopamine transporter (DAT) immunostaining in the NacSh, NacC, and Str. (**E-G**) Quantification of the DAT signal normalized to the anterior commissure intensity for each slice in the NacSh (**E,** 1-way ANOVA F(2,9)=8.46, *P=*0.008557, post hoc CTRL versus CSP *P* =0.142, CTRL versus CSP+2w *P* =0.007974), the NacC (**F,** 1-way ANOVA F(2,9)=5.21, *P* =0.031364, post hoc CTRL versus CSP *P* =0.16, CTRL versus CSP+2w *P* =0.0357), and the Str (**G,** 1-way ANOVA F(2,9)=8.51, *P* =0.008413, post hoc CTRL versus CSP *P* =0.43, CTRL versus CSP+2w *P* =0.008123) in control (black), CSP (blue) or CSP+2w (green). Data are represented as mean±SEM. Individual data are represented as dots when possible. Lines between histograms are used to represent time in a zone or interaction between conditions. *P<0.05.

Figure S2 related to Figure 2


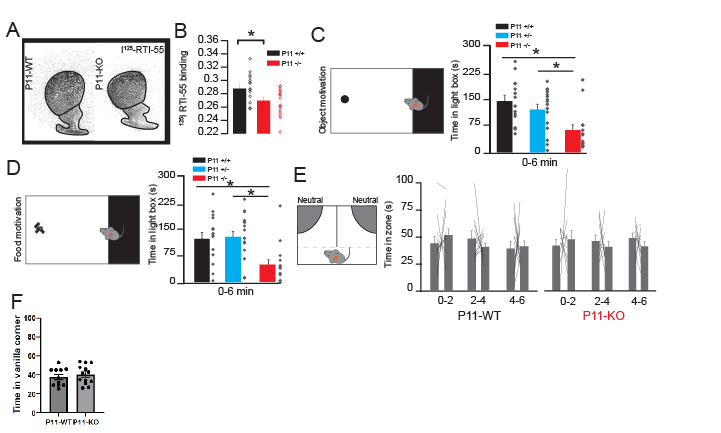


(**A**) Representative images of the autoradiography of DAT binding using the radioligand [^125^I]-RTI-55 in the striatum of P11-WT and P11- KO mice. (**B**) Quantification of the DAT in the striatum (dorsal and ventral) signal subtracted to the background for each slice in P11-WT (black) and P11-KO (red). unpaired t-test *P=*0.003365. (**C**) Time in the light zone during the entire dark-light box test with an unfamiliar object introduced in the light area (0-6 min) for P11-WT (black), P11-HET (blue), and P11-KO (red). 1-way ANOVA F(2,41)=7.0336, *P=*0.002365, post hoc P11-WT versus P11-KO *P=*0.00229, P11-WT versus P11-HET *P=*0.849, P11-HET versus P11-KO *P=*0.0327. (**D**) Time in the light zone during the entire dark-light box test with familiar sugar pellets introduced in the light area (0-6 min) for P11-WT (black), P11-HET (blue), and P11-KO (red). 1-way ANOVA F(2,41)=7.38, *P=*0.001824, post hoc P11-WT versus P11-KO *P=*0.00882, P11-WT versus P11-HET *P=*0.98, P11-HET versus P11-KO *P=*0.0033. (**E**) Time spent in the neutral (grey) zone during habituation of the emotional discrimination test for each segment of 2 minutes (0-2, 2-4, and 4-6 min) for P11-WT and P11-KO. paired t-tests, *P>*0.05. (**F**) Animals tested in the open field with a corner paired with Vanilla extract do not show any olfactory discrimination impairment (unpaired t-test P>0.05). Data are represented as mean±SEM. Individual data are represented as dots when possible. Lines between histograms are used to represent time in a zone or interaction between conditions. *P<0.05.


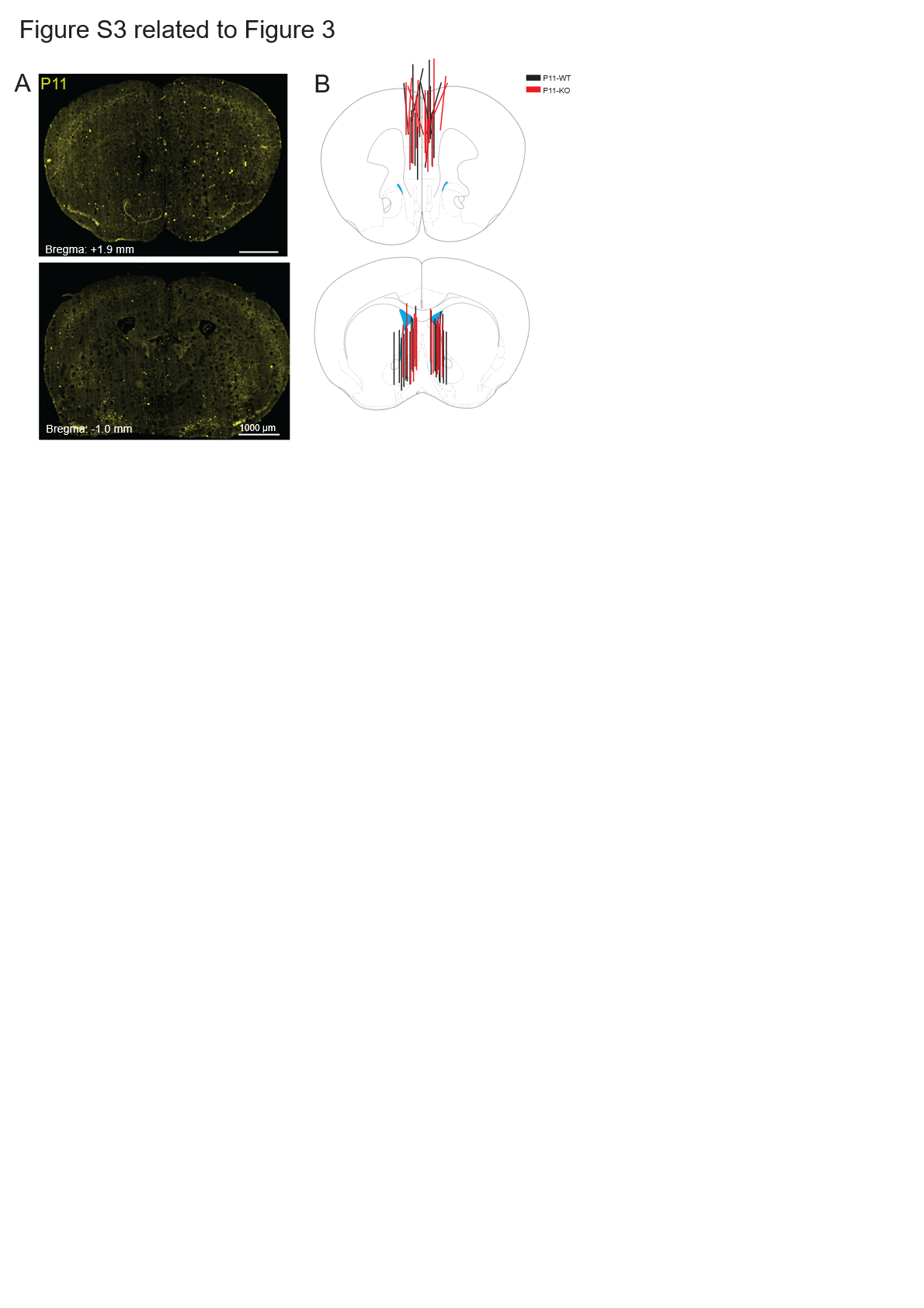


(**A**) RNAscope staining of P11 in the mPFC (top) and the basal forebrain (bottom). (**B**) Reconstructed track of the cannula in P11-WT (black) and P11-KO (red) during *in vivo* recording of mPFC (top) and Nac (bottom) LFP.


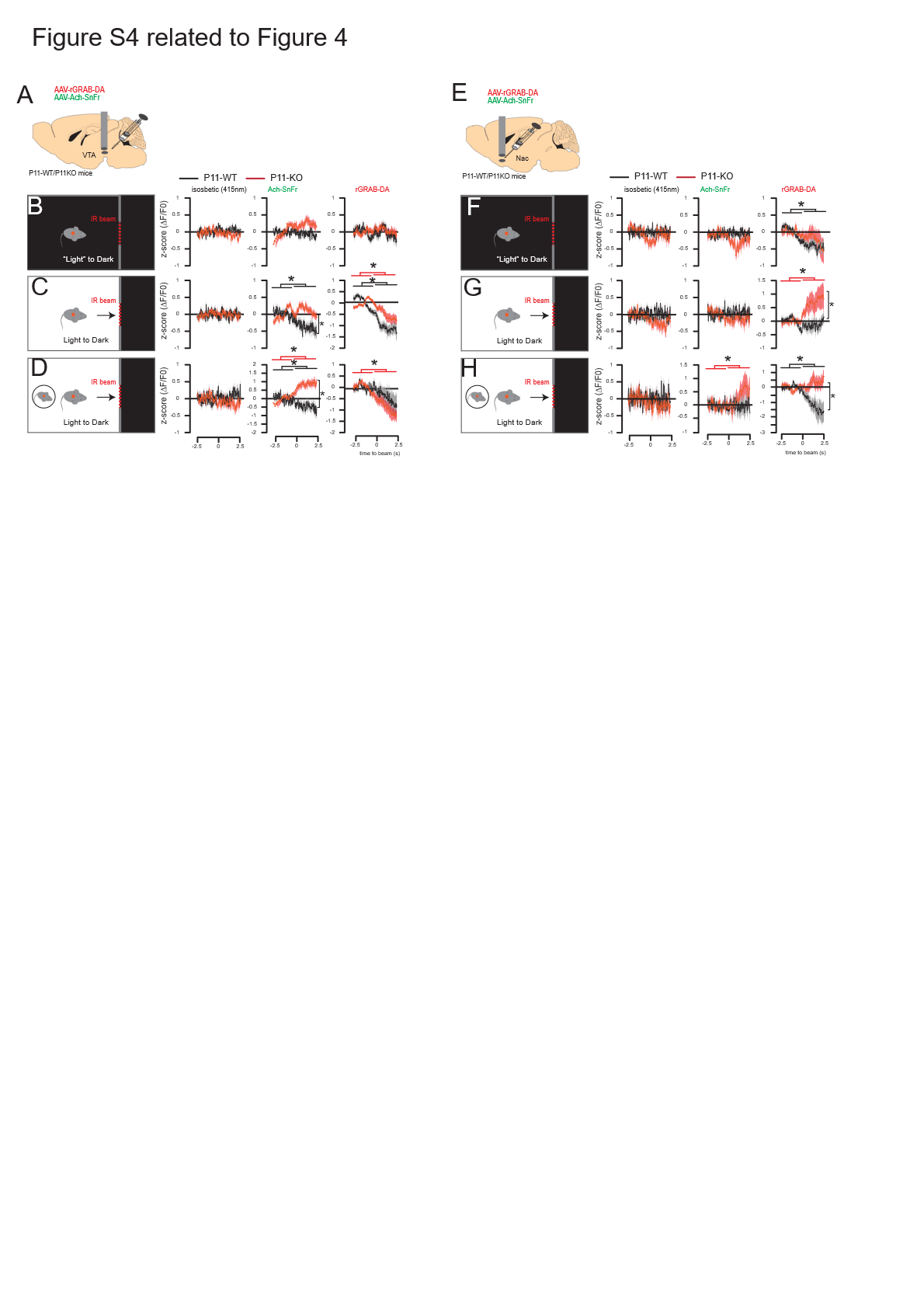


Injection of dopamine- (AAV-rGRAB-DA) and acetylcholine-sensor (AAV-Ach-SnFr) virus in the VTA (**A-D**) or the Nac (**E-H**) of P11-WT (black) or P11-KO (red) mice allow recording of dopamine and acetylcholine release in medial VTA or Nac. (**B-D**) Variation of the GFP-isosbestic control channel (left), acetylcholine (middle) and dopamine release (right panel) in a 5s windows surrounding exit the light area in a light OFF condition (**B,** Ach-sensor: paired t-tests P11-WT *P=*0.1521, P11-KO *P=*0.1201; genotype unpaired t-test P>0.05; DA-sensor; P11-WT *P=*0.4283, P11-KO *P=*0.6249; genotype unpaired t-test P>0.05), light ON (**C,** Ach-sensor: paired t-tests P11-WT *P=*2.10^-5 P11-KO *P=*0.113; genotype unpaired t-test P=4.10^-5; DA-sensor; P11-WT *P=*2.10^-7, P11-KO *P=*0.0016; genotype unpaired t-test P>0.05) and with a conspecific of the same sex animal in the light area (**D,** Ach-sensor: paired t-tests P11-WT *P=*5.10^-7, P11-KO *P=*4.10^-6; genotype unpaired t-test P=9.10^-3; DA-sensor; P11-WT *P=*0.18, P11-KO *P=*0.0001, genotype unpaired t-test P>0.05) in P11-WT (black) and P11-KO (red) mice in the medial VTA. (**F-H**) Variation of the GFP-isosbestic control channel (left), acetylcholine (middle), and dopamine release (right panel) in a 5s windows surrounding exit the light area in a light OFF condition (**F,** Ach-sensor: paired t-tests P11-WT *P=*0.229, P11-KO *P=*0.035; genotype unpaired t-test P>0.05; DA-sensor; P11-WT *P=*1.10^-3, P11-KO *P=*0.352; genotype unpaired t-test P>0.05), light ON (**G,** Ach-sensor: paired t-tests P11-WT *P=*0.143, P11-KO *P=*0.244; genotype unpaired t-test P>0.05; DA-sensor; P11-WT *P=*0.428, P11-KO *P=*0.027; genotype unpaired t-test P=0.028) and with a conspecific of the same sex animal in the light area (**H,** Ach-sensor: paired t-tests P11-WT *P=*0.959, P11-KO *P=*0.004; genotype unpaired t-test P>0.05; DA-sensor; P11-WT *P=*0.016, P11-KO *P=*0.456, genotype unpaired t-test P=1.10^-7) in P11-WT (black) and P11-KO (red) mice in the Nac.


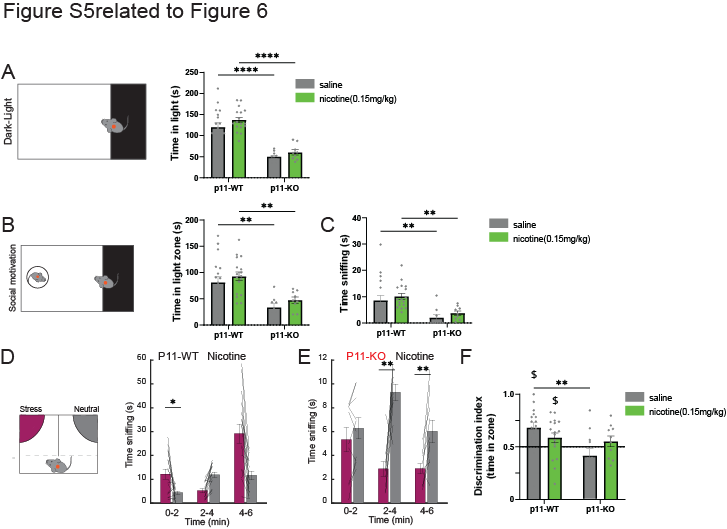


(**A**) Time in the light zone during the entire dark-light box test (0-6 min) 30min after administration of nicotine (green) or saline (grey) in P11-WT or P11-KO (2-way RM-ANOVA genotype effect F(1,24)=45.37, P<0.0001, treatment effect F(1,24)=3.837, P=0.0619, interaction F(1,24)=0.1926, P=0.6647). (**B**) Time in the light zone during the social motivation test (2-way RM-ANOVA genotype effect F(1,24)=23.16, P<0.0001, treatment effect F(1,24)=1.355, P=0.702, interaction F(1,24)=0.011, P=0.9156). (**C**) Time spent sniffing the conspecific stimulus during the social motivation test (2-way RM-ANOVA genotype effect F(1,24)=0.1415, P=0.001, treatment effect F(1,24)=1.09, P=0.3063, interaction F(1,24)=0.0038, P=0.951). (D-E) Time spent in the stress (purple) or neutral (grey) zone during the emotional discrimination test for each segment of 2 minutes (0-2, 2-4, and 4-6 min) in P11-WT (paired t-tests time in zone 0-2 min *P*=0.0056, 2-4 min *P*=7.10^-6, 4-6 min *P*=0.0002, 0-6 *P*=0.0029) and P11-KO (zone 0-2 min *P=*0.1131, 2-4 min *P=*0.0003, 4-6 min *P=*0.0046, 0-6 min *P=*3.10^-5) following administration of nicotine. (F) Discrimination index of the time sniffing the stress and neutral in P11-WT and P11-KO following saline or nicotine administration (2-way RM-ANOVA genotype effect F(1,24)=31.20, P<0.0001, treatment effect F(1,24)=1.841, P=0.6437, interaction F(1,24)=0.22, P=0.637). Data are represented as mean±SEM. Individual data are represented as dots when possible. Lines between histograms are used to represent time in a zone or interaction between conditions. *P<0.05. $ P<0.05 in comparison with 0.5 DI.


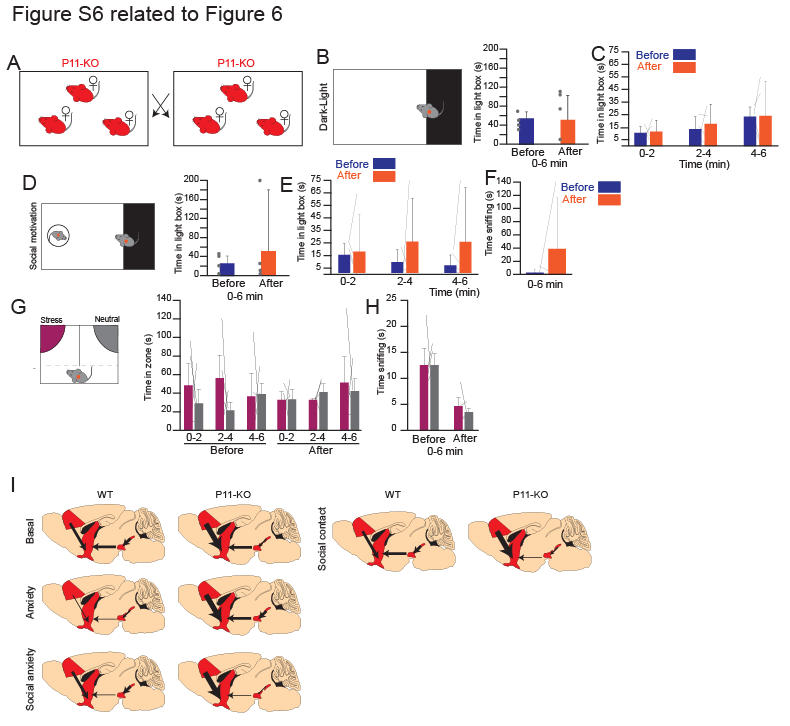


(**A**) Schematic representation of the P11-KO shuffle protocol. (**B**) Time in the light zone during the entire dark-light box test (0-6 min) before (blue) and after (orange) P11-KO shuffle protocol. paired t-test *P=*0.8389. (**C**) Time in the light zone during each segment of 2 minutes of the dark-light box test (0-2, 2-4, and 4-6 min) before (blue) and after (orange) P11-KO shuffle protocol. 2-way ANOVA mixing effect F(1,18)=0.106, *P*=0.74, time effect F(2,18)=1.58, *P*=0.23, interaction, F(2,18)=0.03499, *P*=0.9656. (**D**) Time in the light zone during the entire dark-light box test (0-6 min) with a WT-conspecific introduced in the light area before (blue) and after (orange) P11-KO shuffle protocol. paired t-test *P=*0.5821. (**E**) Time in the light zone during each segment of 2 minutes of the dark-light box test with a WT-conspecific introduced in the light area (0-2, 2-4, and 4-6 min) before (blue) and after (orange) P11-KO shuffle protocol. 2-way ANOVA mixing effect F(1,18)=1.35, *P*=0.2587, time effect F(2,18)=0.006149, *P*=0.9938, interaction, F(2,18)=0.21, *P*=0.8099. (**F**) Time sniffing the WT stimulus during the entire dark-light box test with a WT-conspecific introduced in the light area (0-6 min) before (blue) and after (orange) P11-KO shuffle protocol. unpaired t-test *P=*0.4239. (**G**) Time spent in the stress (purple) or neutral (grey) zone during the emotional discrimination test for each segment of 2 minutes (0-2, 2-4, and 4-6 min) before (paired t-tests time in zone 0-2 min *P*=0.059, 2-4 min *P*=0.645, 4-6 min *P*=0.95, 0-6 *P*=0.48) and after (zone 0-2 min *P=*0.189, 2-4 min *P=*0.127, 4-6 min *P=*0.63, 0-6 min *P=*0.68) P11-KO shuffle protocol. (**H**) Time spent sniffing the stress (purple) or neutral (grey) stimulus in the emotional discrimination (0-6 min) before (0-2 min *P*=0.956, 2-4 min *P*=0.40, 4-6 min *P*=0.55, 0-6 min *P*=0.62) and after (0-2 min *P=*0.89, 2-4 min *P=*0.29, 4-6 min *P=*0.82; 0-6 min *P=*0.50) P11-KO shuffle protocol. (**I**) Schematic representation of the effect of anxiety, social motivation, social interaction, and MDMA in dopamine, acetylcholine, and corticoaccumbal pathway in WT (left) and P11-KO (right). Data are represented as mean±SEM. Individual data are represented as dots when possible. Lines between histograms are used to represent time in a zone or interaction between conditions. *P<0.05.
